# Single-cell RNA sequencing distinctly characterizes the wide heterogeneity in pediatric mixed phenotype acute leukemia

**DOI:** 10.1186/s13073-023-01241-z

**Published:** 2023-10-16

**Authors:** Hope L. Mumme, Sunil S. Raikar, Swati S. Bhasin, Beena E. Thomas, Taylor Lawrence, Elizabeth P. Weinzierl, Yakun Pang, Deborah DeRyckere, Chuck Gawad, Daniel S. Wechsler, Christopher C. Porter, Sharon M. Castellino, Douglas K. Graham, Manoj Bhasin

**Affiliations:** 1 Aflac Cancer and Blood Disorders Center, Children Healthcare of Atlanta, Atlanta, GA USA; 2 Department of Biomedical Informatics, Emory University, Atlanta, GA USA; 3 Department of Pediatrics, Emory University, Atlanta, GA USA; 4 Department of Pathology and Laboratory Medicine, Children’s Healthcare of Atlanta, Atlanta, GA USA; 5 Department: Pediatrics - Hematology/Oncology, Stanford University, Stanford, CA USA

**Keywords:** Mixed phenotype acute leukemia, Single-cell RNA sequencing, Tumor microenvironment

## Abstract

**Background:**

Mixed phenotype acute leukemia (MPAL), a rare subgroup of leukemia characterized by blast cells with myeloid and lymphoid lineage features, is difficult to diagnose and treat. A better characterization of MPAL is essential to understand the subtype heterogeneity and how it compares with acute myeloid leukemia (AML) and acute lymphoblastic leukemia (ALL). Therefore, we performed single-cell RNA sequencing (scRNAseq) on pediatric MPAL bone marrow (BM) samples to develop a granular map of the MPAL blasts and microenvironment landscape.

**Methods:**

We analyzed over 40,000 cells from nine pediatric MPAL BM samples to generate a single-cell transcriptomic landscape of B/myeloid (B/My) and T/myeloid (T/My) MPAL. Cells were clustered using unsupervised single-cell methods, and malignant blast and immune clusters were annotated. Differential expression analysis was performed to identify B/My and T/My MPAL blast-specific signatures by comparing transcriptome profiles of MPAL with normal BM, AML, and ALL. Gene set enrichment analysis (GSEA) was performed, and significantly enriched pathways were compared in MPAL subtypes.

**Results:**

B/My and T/My MPAL blasts displayed distinct blast signatures. Transcriptomic analysis revealed that B/My MPAL profile overlaps with B-ALL and AML samples. Similarly, T/My MPAL exhibited overlap with T-ALL and AML samples. Genes overexpressed in both MPAL subtypes’ blast cells compared to AML, ALL, and healthy BM included *MAP2K2* and *CD81*. Subtype-specific genes included *HBEGF* for B/My and *PTEN* for T/My. These marker sets segregated bulk RNA-seq AML, ALL, and MPAL samples based on expression profiles. Analysis comparing T/My MPAL to ETP, near-ETP, and non-ETP T-ALL, showed that T/My MPAL had greater overlap with ETP-ALL cases. Comparisons among MPAL subtypes between adult and pediatric samples showed analogous transcriptomic landscapes of corresponding subtypes. Transcriptomic differences were observed in the MPAL samples based on response to induction chemotherapy, including selective upregulation of the IL-16 pathway in relapsed samples.

**Conclusions:**

We have for the first time described the single-cell transcriptomic landscape of pediatric MPAL and demonstrated that B/My and T/My MPAL have distinct scRNAseq profiles from each other, AML, and ALL. Differences in transcriptomic profiles were seen based on response to therapy, but larger studies will be needed to validate these findings.

**Supplementary Information:**

The online version contains supplementary material available at 10.1186/s13073-023-01241-z.

## Background

Mixed phenotype acute leukemia (MPAL) is a rare subtype of acute leukemia, accounting for 2%–3% of all newly diagnosed pediatric leukemia cases, with blasts expressing markers of both the lymphoid and myeloid lineage [1, 2]. Antigen expression patterns vary greatly among different MPAL cases, and given the wide phenotypic variability, the diagnostic criteria for MPAL have continued to evolve over the past decades. The European Group for the Immunological Characterization of Leukemias (EGIL) and the World Health Organization (WHO) criteria are the two MPAL classification systems primarily used; however, despite both systems relying on immunophenotype characterization, there remain significant differences in their definitions [1–5]. Given the frequent changes and relative subjectivity in diagnostic criteria, it is extremely difficult to interpret previously published MPAL literature. Reported survival outcomes for MPAL have ranged between 36% and 80%; however, since patients with an MPAL phenotype were excluded from frontline clinical trials until recently, all available treatment and outcome data for MPAL is retrospective. While most patients with MPAL respond to acute lymphoblastic leukemia (ALL) directed therapy [2, 4], there is no clear consensus on how to treat this heterogeneous disease.

The lack of standardized treatment regimens specifically tailored for MPAL is compounded by fluid diagnostic criteria for classifying MPAL and its subtypes. Current classification systems divide MPAL into two broad categories, B/myeloid (B/My) MPAL and T/myeloid (T/My) MPAL [6]. Despite these differences in classification and the wide phenotypic diversity, current treatment approaches have typically considered MPAL to be a single entity with providers primarily choosing between ALL vs. AML regimens, and not considering specific subtypes. Two recent large MPAL genomic studies, one in pediatric patients and one in adults, have shown that B/My and T/My MPAL have distinct genomic signatures [7, 8], thus suggesting that different approaches may be necessary to treat MPAL subtypes. Evolving data has also suggested greater overlap between T/My MPAL and early T-cell precursor ALL (ETP-ALL), a subset of T-ALL that expresses myeloid/stem cell markers not considered to be myeloid lineage defining, hence do not meet the criteria of T/My MPAL. While initial studies showed ETP-ALL had better success with AML-type regimens, more recent data has suggested improved outcomes with ALL regimens, despite higher rates of induction failure [8–10]. Thus, understanding the biological differences between T/My MPAL and ETP-ALL is critically important to design better therapeutic options. Furthermore, two large retrospective cohorts have now shown that the early response to ALL therapy is critical in terms of overall MPAL prognosis, especially in B/My MPAL [8], with patients having positive measurable/minimal residual disease (MRD) at the end of induction (EOI) having significantly poorer outcomes [4, 11]. Analyzing the biology and understanding the similarities and differences is thus critical for improving outcomes in this rare high-risk leukemia. Thus, a more in-depth analysis of MPAL biology is essential to determine effective treatments for this unique disease.

Single-cell RNA sequencing (scRNAseq) analysis has revolutionized cancer research by revealing cell types, pathways, and cellular interactions that play a critical role in malignant cell progression and response to therapy [12, 13]. Identifying changes in cellular and molecular profiles is critical for identifying novel targets for diagnosis, risk assessment, and clinical outcomes. Single-cell profiling can be invaluable for deep characterization, given the wide phenotypic and genomic diversity seen in MPAL. Only one single-cell study of MPAL has been previously reported, using six MPAL samples from adults (one B/My and five T/My MPAL) [14]. Here, we present for the first time scRNAseq profiling of nine pediatric MPAL samples, along with a comparative analysis with previously generated scRNAseq datasets from pediatric AML and ALL samples including ETP-ALL, young adult healthy bone marrow (BM) samples, and adult MPAL samples [14–18]. Additionally, we performed a comparative analysis among the diagnostic MPAL samples based on their response to ALL-directed induction therapy. Our results provide the initial framework of the pediatric MPAL single-cell signature and support utilizing scRNAseq analysis for further characterization of the MPAL blasts and marrow landscape.

## Methods

### Bone marrow samples

Viably frozen de-identified pediatric MPAL BM samples were obtained from the Leukemia/Lymphoma Biorepository at the Aflac Cancer and Blood Disorders Center at Children’s Healthcare of Atlanta (CHOA). Patients and/or their legal guardian(s) provided written informed consent that permitted the use of biological material in accordance with a protocol that was approved by the CHOA Institutional Review Board (IRB) (Protocol #00034535). The immunophenotypic characterization of MPAL was made according to the updated World Health Organization (WHO) 2022 MPAL criteria [5]. BM samples were collected at the initial presentation as part of routine diagnostic evaluation. Corresponding available clinical cytogenetic, molecular, and flow cytometric data characterizing these cases as well as treatment information for each case was also obtained. ScRNAseq analysis was performed on seven MPAL patient samples, four of which were classified as B/My MPAL and three T/My MPAL based on their immunophenotype. Additionally, we also incorporated raw gene expression data from two T/My MPAL patients from the Single-cell Pediatric Cancer Atlas (ScPCA) [15] for characterizing the landscape of pediatric MPAL. To perform comparative analysis, we utilized single-cell datasets from other pediatric leukemias, including AML (*n* = 15) and T-ALL (*n* = 11), obtained from previous or ongoing studies in our lab [17, 18]. Additionally, we incorporated publicly available scRNAseq datasets from young adult and pediatric healthy BM (*n* = 9), pediatric B-ALL (*n* = 7), and AML (*n* = 8) samples as well as adult MPAL samples (*n* = 6) [14–16, 19].

### Clinical, pathological, and treatment characteristics of MPAL cases

A total of nine pediatric MPAL cases were included in this analysis, eight of which were collected at the time of initial diagnosis and one at relapse. Based on their immunophenotype, four cases were classified as B/My MPAL and five were categorized as T/My MPAL. The mean age for the study population was 13.4 years (range: 8.7–15.8 years) and included seven males and two females. Overall clinical characteristics of the study population are summarized in Table 1, and detailed individual case characteristics are described in Additional file 1: Table S1. Available clinical-grade molecular, cytogenetic, and flow cytometry-based characterizations of these leukemias are summarized in Table 2 and Additional file 1: Table S2. Cytogenetic analysis revealed that among the B/My MPAL cases, one case had a *KMT2A*-R with t(4;11)(q21;q23) (M1), and another one was positive for t(9;22) (q34;q11.2) and monosomy 7 (M3). Among the T/My MPAL cases, translocations seen included t(3;15)(p21;q24) (M2), t(2;3)(p15;q26.2) (SCPCS000220), and t(7;14)(q21;q32) (SCPCS000230). Molecular findings in T/My MPAL cases included FLT3-ITD with elevated allelic ratio of 0.15 (M2), mutations in *NRAS*, *NOTCH1*, *ETV6*, and *MED12* (M4), and alterations in *KDM6A*, *RUNX1*, *SUZ12*, *TP53*, *JAK3*, and *ASXL1* (M6). Flow cytometric immunophenotyping showed a heterogeneous pattern of blast populations within individual MPAL cases. Some cases had separate distinct blast populations (M3, M5, SCPCS000230), whereas other cases had minor subsets within the larger blast population with unique surface profiles (M1, M2, M4, M6, M7, SCPCS000220). Several T/My MPAL samples had blasts with ETP-ALL-like immunophenotypic features (M2, M4, M6). Available detailed flow cytometric immunophenotypic characterization on peripheral blood blasts and bone marrow blasts is summarized in Additional file 1: Table S2. Seven patients included in this analysis received a Children’s Oncology Group (COG) based ALL-directed induction therapy. Specific regimens are listed in Additional file 1: Table S1. At the end of induction (EOI), out of the four B/My MPAL samples, one was negative for MRD (M1), two were positive for MRD (M3, M5), and one had induction failure (M7). Of the three de novo T/My MPAL samples that received ALL induction therapy, one was MRD negative (M2), one was MRD positive at EOI (M4) and one had induction failure (M6). Overall, four of the nine patients had relapsed/refractory disease and six were alive at the last follow-up (Table 1 and Additional file 1: Table S1).

**Table 1:** Characteristics of pediatric MPAL patients analyzed in this study. Patient characteristics and clinical information with sex, age at disease diagnosis/relapse (Dx/Rel), white blood cell (WBC) count at Dx/Rel, peripheral blood blast percentage, bone marrow blast percentage, timepoint of sample collection, response to ALL-based induction therapy, relapsed/refractory disease, and patient status information. Response to ALL-based induction therapy is shown as minimal residual disease (MRD) positive (> 0.01%), MRD negative (< 0.01%), and induction failure (> 5%)

Characteristic	All MPAL (*n* = 9)	B/My MPAL (*n* = 4)	T/My MPAL (*n* = 5)
Sex			
Male	7 (78%)	3 (75%)	4 (80%)
Female	2 (22%)	1 (25%)	1 (20%)
Age at presentation (years)			
Mean (range)	13.4 (8.7–15.8)	14.6 (13.7–15.1)	12.5 (8.7–15.8)
< 10	1 (11%)	0 (0%)	1 (20%)
≥ 10	8 (89%)	4 (100%)	4 (80%)
WBC at presentation (× 10^3^/uL)			
Mean (range)	65.5 (7.1–180.1)	56.1 (12.8–134.6)	73 (7.1–180.1)
< 50	6 (67%)	3 (75%)	3 (60%)
≥ 50	3 (33%)	1 (25%)	2 (40%)
Peripheral blood blast %			
Mean (range)	59.5 (5.0–95.7)	65.0 (31.8–95.7)	54.0 (5.0–73.5)
Bone marrow blast %			
Mean (range)	82.6 (40.2–97.7)	92.4 (82.0–97.7)	72.8 (40.2–96.4)
Timepoint of sample collection			
Initial diagnosis (Dx)	8 (89%)	4 (100%)	4 (80%)
Relapse	1 (11%)	0 (0%)	1 (20%)
Response to ALL-based induction therapy (*n* = 7)			
MRD negative	2/7 (29%)	1/4 (25%)	1/3 (33%)
MRD positive	3/7 (43%)	2/4 (50%)	1/3 (33%)
Induction failure	2/7 (29%)	1/4 (25%)	1/3 (33%)
Relapsed/refractory disease			
Yes	4 (44%)	1 (25%)	3 (60%)
No	5 (56%)	3 (75%)	2 (40%)
Patient status			
Alive	6/9 (67%)	3 (75%)	3 (60%)
Deceased	3/9 (33%)	1 (25%)	2 (40%)

*MPAL* mixed phenotype acute leukemia, *B/My* B/myeloid, *T/My* T/myeloid, *WBC* white blood count, *MRD* minimal residual disease

**Table 2:** Clinical-grade molecular and cytogenetic information for MPAL patients in this study. For each sample and corresponding sample ID, the diagnosis subtype, karyotype, fluorescence in situ hybridization (FISH) results, microarray single nucleotide polymorphism (SNP) copy number (CN) results, next-generation sequencing results, and FLT3-ITD/FLT3 mutation presence are listed

Sample ID	Diagnosis subtype	Karyotype	FISH results	Microarray SNP-CN analysis	Next-generation sequencing	FLT3-ITD/FLT3 mutation
M1	B/My MPAL	46,XX,t(4;11)(q21;q23)[3]/46,sl,i(7)(q10)[9]/46,XX	11q23 rearrangement	No copy number abnormalities detected	Not available	Negative
M2	T/My MPAL	46,XY,t(3:15)(p21;q24)[8]/46,XY[22]	Negative	Gain 2q, Loss 3p containing SETD2, Loss 15q, Loss 13q, CN-LOH 11p	CN-LOH 11p	FLT3-ITD positive, allelic ratio 0.15
M3	B/My MPAL	46,XY,t(9;22)(q34;q11.2)[18]/44,sl,-3,-7[2]	t(9;22), monosomy 7	Loss of 3 and 7	Not available	Negative
M4	T/My MPAL	44–47,XY,add(3)(p25),del(3)(q12),-4, + 6,add(6)(q13),der(8)t(8;11)(q24.1;q12),add(9)(p13),del(9)(p13),del(13)(q14),add(16)(p13.3), ish del(5)(q31.2q31.2)(EGR1-)[cp3]/46,XY[65]	Negative	Not available	NRAS (G12V), NOTCH1 (S2341fs), ETV6 (R369fs), MED12 (R621*)	Negative
M5	B/My MPAL	46,XY [30]	Negative	Focal loss of portion of IKZF1 gene (7p), Focal gain of 11q with a breakpoint within DCUN1D5 gene	Not available	Negative
M6	T/My MPAL	45 ~ 46,XY,del(3)(q25),add(5)(q31),del(6)(q21q25),t(10;11)(q26;q13),add(11)(p15),del(13)(q12q14),del(17)(p11.2), + mar[cp9]/46,XY[12]	Negative	Loss of 5q,1q, 2q, 6p, 11q containing BIRC3 and ATM, 13q containing RB1, and 17p containing TP53, and CN-LOH of 2q	KDM6A (Y1354*), RUNX1 (duplication exon 4), SUZ12 (H466fs*23), TP53 (G245S), JAK3 (L857P), ASXL1 (G646fs)	Negative
M7	B/My MPAL	46,XY,del(2)(p11.2p13)[6]/46,XY[22]	Negative	Loss of 2p, loss of 9p contianing MLLT3 with a region of homozygous loss containing CDKN2A/B, CN-LOH of 9p containing AK3, JAK2, PAX5	Not available	Negative
SCPCS000220	T/My MPAL	46,XX,t(2;3)(p15;q26.2)[cp5]/46,idem,del(20)(q11.2q13.3)[cp5]	The t(2;3) observed here is consistent with a translocation involving MECOM (EVI1)	Not available	Not available	Negative
SCPCS000230	T/My MPAL	46,XY,t(7;14)(q21;q32)[21]	t(7;14), Negative MLL, IGH, FGFR, and FIP1L gene rearrangement	Not available	Not available	Negative

*MPAL* mixed phenotype acute leukemia, *B/My* B/myeloid, *T/My* T/myeloid, *FISH* fluorescence in situ hybridization, *SNP* single nucleotide polymorphism, *CN* copy number

### Single-cell RNA sequencing and analysis of MPAL samples

ScRNAseq libraries were prepared from viably revived BM samples using anti-human hashtag antibodies (Biolegend) and Chromium single-cell 3′v3 reagent kits (10x  Genomics). Sequencing was performed using NextSeq 500 high output and Novaseq S4 kits (Illumina). The fastq files were analyzed using Cell Ranger version 7.0.0 [20] for demultiplexing, alignment to the human genome (hg38), and generation of gene-count matrices for further bioinformatics analysis.

### Single-cell profiling data from other leukemias and healthy bone marrow

For comparative analysis of MPAL with other pediatric leukemias, we utilized single-cell datasets generated in our lab for other leukemias: AML (*n* = 15), and T-ALL (*n* = 11) [17, 18, 21, 22]. The T-ALL data contains both samples with ETP-ALL-like features (ETP-ALL and near ETP-ALL, *n* = 4) and non-ETP T-ALL (*n* = 7). Data were generated and processed using the uniform approach briefly described in the following paragraph and previously utilized [17, 18]. We also used publicly available datasets, downloaded via the Gene Expression Omnibus (GEO) portal (GSE154109), for comparative analysis [16, 23]. This dataset contained pediatric B-ALL (*n* = 7), pediatric AML (*n* = 8), and young adult healthy BM (*n* = 4) samples. Additionally, we also obtained young adult and pediatric healthy BM samples (*n* = 5) from ScPCA [15] and GEO (GSE132509) [19, 24] for comparative analysis with MPAL. Furthermore, we conducted a comparative analysis of adult and pediatric MPAL using one B/My and five T/My MPAL adult scRNAseq data obtained from a recently published study [14, 25].

### Single-cell profiling data analysis

**Fig. 1:**
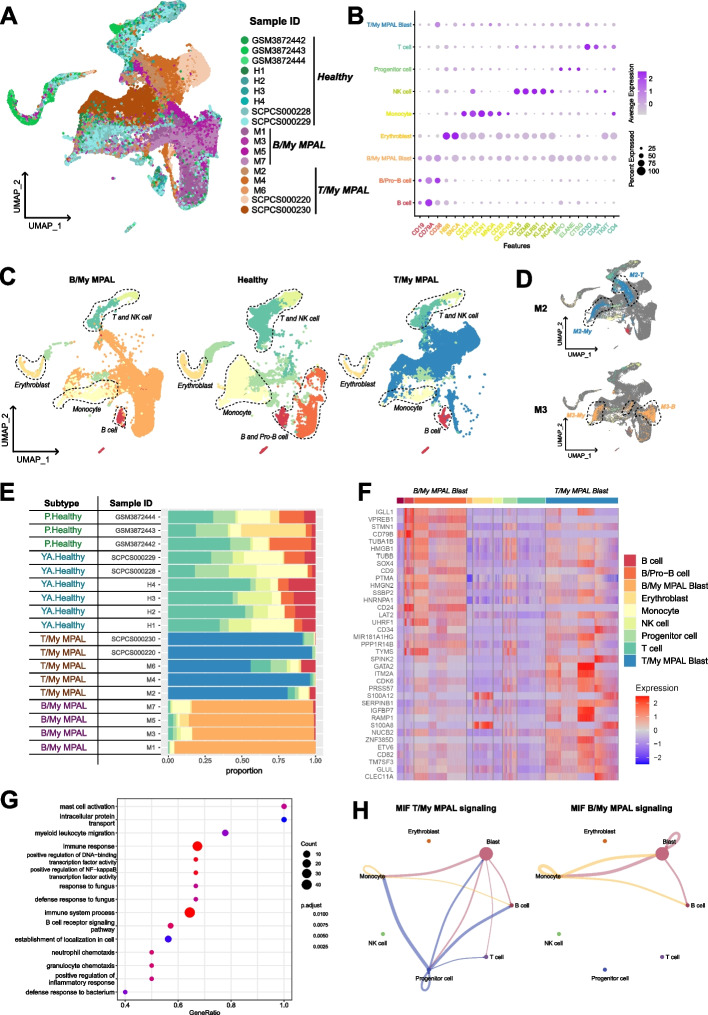
Comparative analysis of mixed phenotype acute leukemia (MPAL) samples single-cell landscape with healthy bone marrow (BM). **A** UMAP showing the profile of MPAL and healthy samples (*n* = 67,024 cells), colored based on the individual sample. **B** Dot plot showing expression of canonical cell markers used to annotate clusters on the *X*-axis and final cell type labels on the *Y*-axis. **C** Split UMAP based on clinical groups (i.e., B/My MPAL, T/My MPAL, Healthy) to visualize cellular clusters associated with specific clinical groups. Dotted lassos highlight the locations of the immune cell populations. **D** UMAP highlighting the heterogenous blast populations from selected patients. The cell types from M2 (T/My MPAL) and M3 (B/My MPAL) are highlighted on the UMAP. The major blast populations are shown (lassoed) for each sample: M2-My and M2-T, and M3-My and M3-B. **E** Table and bar plot with cell type distributions, disease subtype, MRD status after treatment, and clinical outcomes. **F** Heatmap showing top 20 overexpressed genes in B/My MPAL and T/My MPAL blast cells. DEGs were identified by comparing the profile of B/My or T/My MPAL blast cells and healthy immune cells based on fold change and adjusted *P*-values (i.e., average log2FC > 0.25 and adjusted *p*-value < 0.05). The top 20 genes were selected based on the highest fold change. **G** Gene ontology enrichment results for the overexpressed genes (average log2FC > 0.25 and adjusted *p*-value < 0.05) in MPAL blasts compared to progenitor cells in healthy BM samples. The gene ontology analysis was performed using clusterProfiler package from R/Bioconductor using Biological Process GO categories. The Biological Process with Benjamini–Hochberg *p*-value < 0.05 is considered significant. The X-axis represents the GeneRatio, which indicates the fraction of MPAL significantly overexpressed genes that can be found in biological gene sets (specifically, GO categories). The size of each dot corresponds to the count of input genes that are present in a particular GO biological category. The color of the dot reflects the adjusted *p*-value obtained from the enrichment analysis. Specifically, pink and blue colors are used to represent the most and least significantly enriched GO terms associated with MPAL significantly overexpressed genes, respectively. **H** Macrophage migration inhibitory factor (MIF) signaling in T/My and B/My MPAL cell types. Signaling was inferred using cellular communication analysis, showing the estimated interactions between cell types in MPAL samples via the ligand (MIF) and receptors (CD74, CXCR4, CD44) expression

**Fig. 2:**
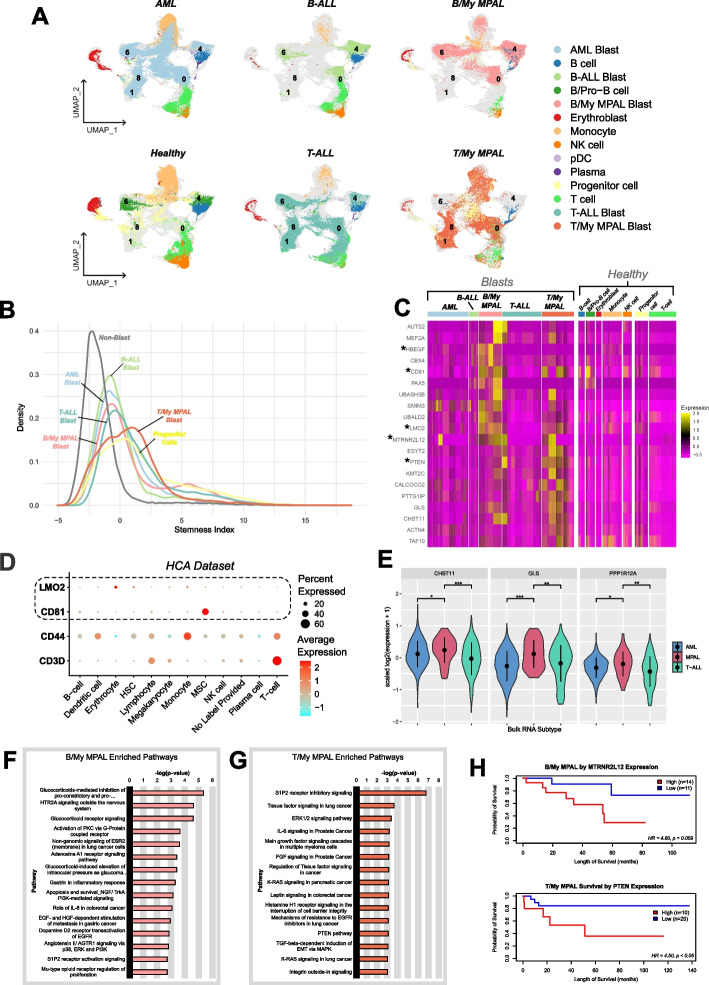
Comparative analysis of mixed phenotype acute leukemia with other acute leukemias. **A** Split UMAP of leukemic and canonical cell types (*n* = 156,489 cells), separated based on leukemia type/subtype (i.e., AML, B-ALL, T-ALL, B/My MPAL, and T/My MPAL) and healthy samples. **B** Density plot showing stemness index distribution of the different blast cells from different acute leukemias including B/My MPAL and T/My MPAL, progenitor cells, and normal immune cells. The stemness index was calculated as the first principal component value of each cell after performing principal component analysis with the expression of the genes in a stem cell signature (Additional file 1: Table S3). **C** Heatmap with the top overexpressed markers for mixed phenotype acute leukemia (MPAL) and subtypes (i.e., B/My MPAL and T/My MPAL). The heatmap also shows the expression of MPAL marker genes in other acute leukemias (i.e., AML, B-ALL, T-ALL), (BM) and healthy immune cells. These markers were filtered to only include genes with low expression in healthy bone marrow cells. Overexpressed genes were identified for MPAL subtypes by comparing the profile of MPAL blast cells versus blast cells from other acute pediatric leukemias (i.e., AML, B-ALL, T-ALL) and healthy BM samples. The MPAL subtype significantly overexpressed genes (average log2FC > 0.25, adjusted *p*-value < 0.05, and pct. expressed > 50%) were further refined by selecting genes with low expression in healthy BM cells from HCA (avg. expression < 0.5). Finally, the top genes for the heatmap were chosen based on their highest average log2FC values. **D** Dot plots showing the expression of two canonical immune cell markers (*CD79A* and *CD3D*) and two MPAL blast cell markers (*CD81* and *LMO2*), to show that these MPAL blast cells markers have low expression in various normal BM cell types and healthy hematopoietic stem cells. The size of the dots refers to the percentage of cells in each cell type cluster expressing the gene and the color represents averaged scaled gene expression level; cyan: low, red: high. X-axis is the cell type, and Y-axis is the genes. The expression of MPAL markers is marked with lasso. **E** Expression of MPAL blast markers in AML, T-ALL, and MPAL bulk RNA-seq data. The Y-axis shows the scaled values of the log2 of the normalized expression plus one, and the X-axis shows different subtypes for the bulk RNA-seq samples. Wilcoxon rank tests were performed to test the difference in expression between MPAL and AML, and MPAL and T-ALL for the three genes shown (*** for *p*-value < 0.001, ** for *p*-value < 0.01, and * for *p*-value < 0.05). **F** The top significantly enriched pathways of the filtered B/My MPAL blast cell marker genes. Each bar represents a significantly enriched pathway as determined using the *P* value (shown on the primary X-axis). The bar plot is sorted by the negative log of the hypergeometric distribution-based *p*-values of the results. The analysis for canonical pathways was performed using the MetaCore platform from Clarivate Inc. **G** The top significantly enriched pathways of the filtered T/My MPAL blast cells marker genes. **H** Kaplan–Meier curves-based survival association analysis of B/My MPAL marker gene, *MTRNR2L12* in B/My MPAL TARGET samples (top) and T/My MPAL marker, *PTEN* in T/My MPAL TARGET samples (bottom). Survival association analysis was performed using the Cox Proportional Hazards Regression Model, with *MTRNR2L12* expression having a hazard ratio of 4.80 (*p* = 0.059) and *PTEN* expression having a hazard ratio of 4.50 (*p* = 0.04), high expression of both genes indicated an association with poor survival

**Fig. 3:**
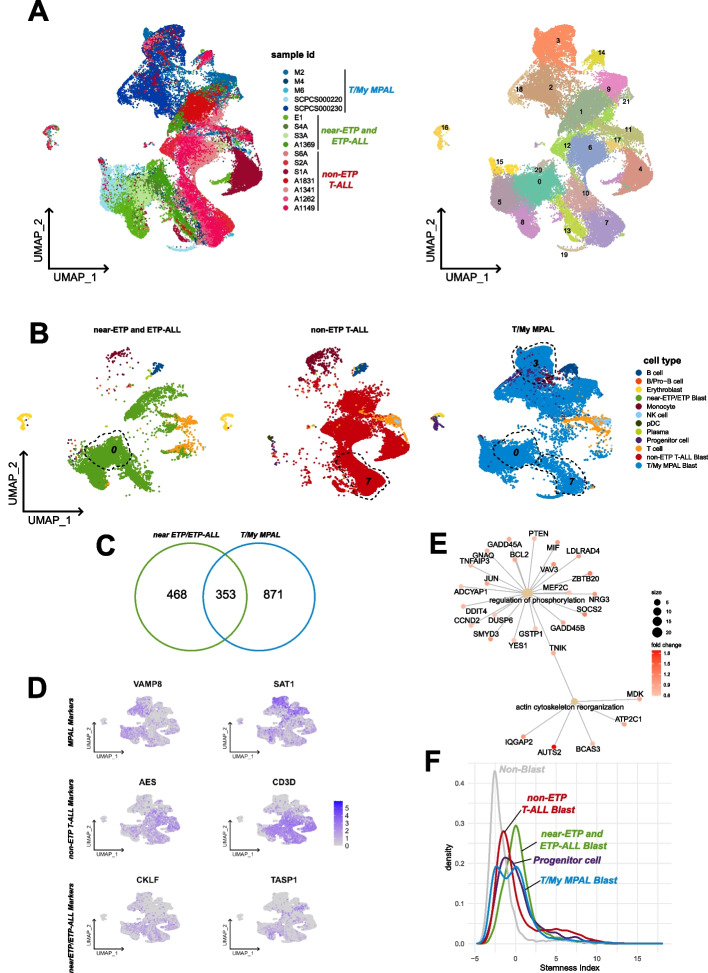
Mapping the single-cell landscape of early T-cell precursor acute lymphoblastic leukemia (ETP-ALL). **A** UMAP clusters of 50,907 cells colored based samples and different ALL (left) including T/myeloid mixed phenotype acute leukemia (T/My MPAL), near-ETP/ETP-ALL, and non-ETP T-ALL. The right side is the UMAP colored by clusters obtained based on K-mean clustering using the Seurat package. **B** Cell type annotations for the three T-Lineage subtypes shown on UMAPs. Clusters with the overlap of cells and transcriptome profiles among different T-ALL subtypes have been lassoed and labeled. **C** Venn diagram analysis to visualize commonly overexpressed genes (average log2FC > 0.25, adjusted *p*-value < 0.05) in T/My MPAL compared to non-ETP T-ALL blast cells, and near-ETP/ETP-ALL compared to non-ETP T-ALL blast cells. **D** Feature map of selected T/My MPAL, non-ETP T-ALL, and near-ETP/ETP-ALL blast cells overexpressed genes. Low and high expressions are shown with gray and purple colors respectively. **E** Gene network plot for enriched GO categories associated with overexpressed near-ETP/ETP-ALL genes. The network nodes have been colored based on fold change in near-ETP/ETP-ALL, and the size of the central dots represents the size of the selected GO category. **F** Density plot showing stemness index distribution of blast cells T/My MPAL, near-ETP/ETP-ALL, non-ETP T-ALL, and non-blast immune cells. The stemness index was calculated as the first principal component value of each cell after performing principal component analysis with the expression of the genes in a stem cell gene set as the features (Additional file 1: Table S3)

**Fig. 4:**
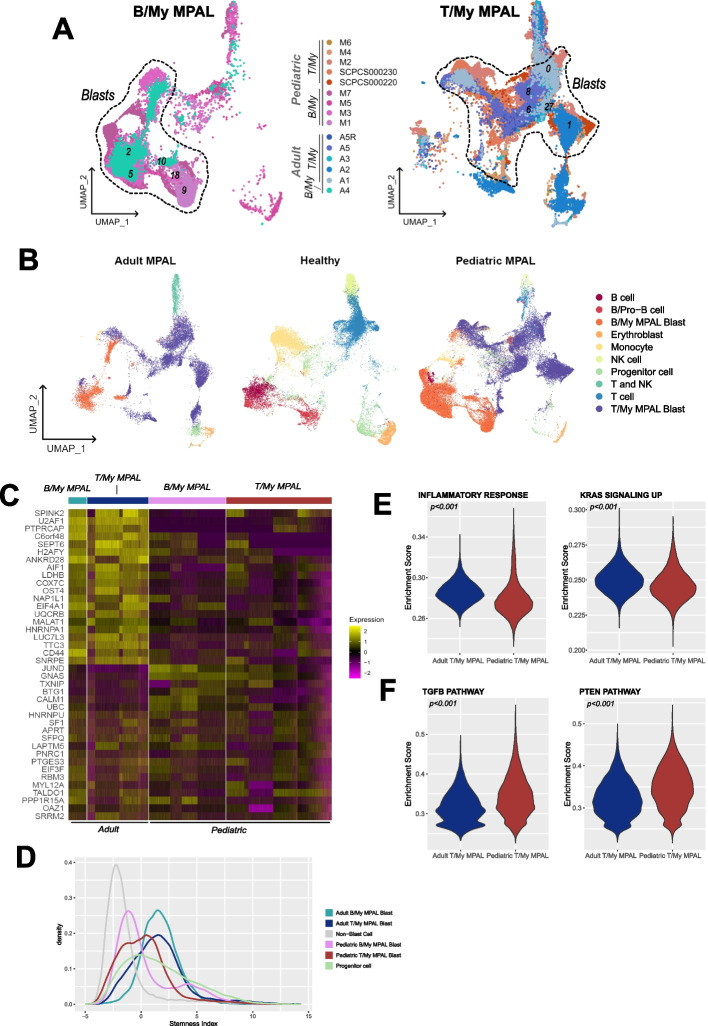
Comparative analysis of pediatric and adult mixed phenotype acute leukemia single-cell landscape. **A** Split UMAP plots of B/My MPAL and T/My MPAL colored based on the respective patient samples. The adult MPAL samples are represented in shades of blue and green, while the pediatric are depicted in shades of red and pink. **B** Comparative visualization of malignant blasts and normal microenvironment cell types in the adult, pediatric, and healthy samples. **C** Heatmap of top genes overexpressed in adult vs. pediatric MPAL blast cells. Genes were identified by performing differential expression analysis selecting genes with average log2FC > 0.25 and adjusted *p*-value < 0.05. The top genes for the heatmap were selected based on average log2FC. Relative gene expression is shown in pseudo color, where purple represents downregulation, and yellow represents upregulation. **D** Density plot showing the distribution of stemness index of different adult and pediatric MPAL subtypes and normal cells. Density plot showing stemness index distribution of the different cell types found in T/My MPAL samples. The stemness index was calculated as the first principal component value of each cell after performing principal component analysis with the expression of the genes in a stem cell gene set as the features (Additional file 1: Table S3). **E** Selected gene sets with significantly higher enrichment (*p*-value < 0.001) in adult T/My MPAL blast cells. **F** Gene sets with higher enrichment (*p*-value < 0.001) in pediatric versus adult T/My MPAL blast cells. The enrichment score was calculated using a single-sample gene set enrichment approach using Hallmark/Biocarta gene sets from the MSigDb H and C2 collections and the significance of differential enrichment was determined using the Wilcoxon rank-sum test

Raw gene-count matrices from samples were merged to generate a raw expression matrix from pediatric leukemias and healthy BM. Cells were filtered based on mitochondrial content and feature count (pct. mitochondrial < 20 and feature count < 5000, > 200). Expression profiles were normalized and scaled using the pre-processing functions in Seurat v4 [26]. To remove the systematic variation in the expression data from various resources, we performed batch correction using the Harmony algorithm version 0.1.1 (with default parameters) for the analysis shown in Figs. 2, 3, 4, and 6, which uses a graph-based approach to model the variation in gene expression across batches [27], or the integration anchors method (with default parameters, 2000 integration features were selected using “vst” method) by Seurat v4, for the analysis shown in Fig. 1, to identify anchor correspondences between data sources to minimize variation [28]. Principal component analysis (PCA) was performed (number of PCs = 30), and batch-corrected expression data was visualized on a low dimensional space via the UMAP approach. The cells were clustered using the K-nearest neighbor graph-based clustering approach (dims = 1:30, resolution = 0.5). Leukemic cells, or blast cells, were annotated by comparing each leukemia set (AML, B-ALL, T-ALL, MPAL) samples with the healthy control BM profile and identified as cells that did not cluster with the healthy control cells. Once the blasts were identified, the non-blast or canonical lymphoid, myeloid, and erythroid lineage cells were annotated based on a combination of automatic annotation using the SingleR package v1.8.1 [29], and manual annotation via known marker genes (Fig. 1B). SingleR is an automatic annotation tool that labels cells based on an external annotated reference, such as the Human Primary Cell Atlas (ERP122984), which we utilized in our analysis [30].

Differential Gene Expression (DEGs) analysis was performed to generate MPAL blast transcriptome signatures by comparing gene expression profiles of MPAL subtype blast cells with healthy cells using the Wilcoxon rank test, with the *FindMarkers* function from Seurat v4 (compared B/My MPAL blast cells versus all healthy cells, repeated for T/My MPAL blast cells). The transcriptome signatures were generated based on fold change and P-value cutoffs (adjusted *p*-value < 0.05, average log2FC > 0.25, and percent cell expression > 50%). The analysis identified transcriptome signatures for MPAL as well as subtypes, i.e., T/My MPAL and B/My MPAL. In addition to identifying genes that are overexpressed in MPAL selectively, we also performed differential expression analysis between MPAL subtypes versus other acute pediatric leukemia (i.e., AML, B-ALL, T-ALL) and healthy BM cells using *FindMarkers* function (adjusted *p*-value < 0.05, average log2FC > 0.25, and percent cell expression > 50%) from Seurat v4 package. Also, in the ETP analysis of Fig. 3, DEGs were identified by comparing T/My MPAL to non-ETP T-ALL blast cells, and near-ETP/ETP-ALL to non-ETP T-ALL blast cells using the *FindMarkers* function (average log2FC > 0.25, adjusted *p*-value < 0.05) from Seurat v4 package. To identify DEGs based on clinical outcomes, we performed differential expression analysis between blasts from MRD outcome groups or Dx-Rel and Dx-Rem blasts for each MPAL subtype with Seurat v4 *FindMarkers* function (average log2FC > 0.25, adjusted *p*-value < 0.05, and percent expressed > 0.7 for Dx-Rem and percent expressed in comparison group < 0.1 for Dx-Rel). In the DEGs analysis, the calculation of the adjusted *p*-value was performed using the Bonferroni correction in the Seurat v4 FindMarkers function.

### Generation of MPAL blast-specific gene dysregulation

Transcriptome signatures generated from the above analysis were systematically compared with normal and stem cell profiles from the human cell atlas (HCA). MPAL blast genes with an average expression of > 0.5 in normal BM or stem cells from the HCA were considered non-specific and filtered out. The analysis resulted in the identification of MPAL blast cells specific overexpressed genes that were further compared with pediatric ALL and AML leukemia blast cells to identify MPAL-specific genes with high potential to be MPAL biomarker candidates (Additional file 2: Fig. S1).

Additional validation of MPAL blast biomarkers was performed using the TARGET bulk RNA-seq dataset [31], which includes AML (*n* = 1199), T-ALL (*n* = 221), B/My MPAL (*n* = 21), and T/My MPAL (*n* = 25) patients. Transcripts per million (TPM) normalized data was obtained from the TARGET portal and log transformed for comparative analysis. The Wilcoxon rank test was performed to compare the expression levels of each MPAL blast biomarker in MPAL versus AML and T-ALL samples. The analysis was conducted using the stat_compare_means function in the ggpubr v0.6.0 package. The corresponding *p*-values are reported.

### Pathway enrichment analysis

MPAL subtype blast-specific pathways were identified by performing pathways and systems biology analysis using the MetaCore platform. The B/My MPAL blast-specific DEGs were identified by comparing the expression between B/My MPAL blasts and other leukemia blasts, as well as with immune cells (Wilcoxon rank test, adjusted *p*-value < 0.05 and average log2FC > 0.25). A similar DEG analysis was performed to identify T/My MPAL blasts dysregulated genes. The DEGs were used for pathways enrichment analysis using the MetaCore platform that contains functions, pathways, and network models derived by systematically exploring peer-reviewed scientific literature and public databases. It calculates statistical significance based on the hypergeometric distribution where the p-value represents how likely the observed association between a specific pathway/function/interactive network and the input dataset would be if it were only due to random chance, by also considering the total number of functions, pathways, and interactive network eligible genes in the dataset and the Reference Set of genes (those which potentially could be significant in the dataset). Focus molecules were identified from the integrated networks based on the degree of connectivity (number of interactions for each gene). Focus hubs with higher degrees of connectivity are considered critical for the maintenance of the networks, suggesting that therapeutic targeting of these focus hubs may elicit the strongest impact. Pathways and networks with a false discovery rate (FDR) < 0.05 (calculated using the Benjamini–Hochberg method) were considered statistically significant.

### Gene set enrichment analysis

In addition to individual gene analysis, gene set enrichment analysis (GSEA) was implemented to determine whether a priori-defined set of genes showed statistically significant, concordant differences between different group comparisons [32]. GSEA can be more powerful than single-gene methods for studying the effects of factors such as MRD in which each set of genes make subtle contributions to have a cumulative effect. Two methods were used for performing GSEA, including (i) the easy single-cell analysis platform for enrichment (escape) R package, version 1.5.1 [33]. Gene sets were obtained from the molecular signature database via the msigdbr package (v7.4.1). Another method (ii), clusterProfiler (v4.6.0) R package [34], was used to perform GSEA with the Gene Ontology (GO) biological processes gene sets to generate enrichment map networks and gene concept enrichment plots. Once the significantly upregulated pathways were identified for each leukemia type, the comparative analysis of pathways resulted in the identification of T/My and B/My MPAL blasts-specific pathways. Gene sets with an adjusted *p*-value < 0.05, determined through the Benjamini–Hochberg correction (clusterProfiler), or the Bonferroni correction (escape), were considered enriched. These GSEA methods were also used to identify adult, pediatric, and induction outcome-specific pathways.

### Cellular communication analysis

Cellular communication analysis was performed using CellChat v1.0.0 [35], with default parameters. CellChat uses ligand-receptor expression to predict intercellular communication among specific signaling pathways. CellChat first calculates the communication probability of each signaling pathway in the CellChatDB ligand-receptor database between cell clusters or cell types within a group (B/My vs. T/My MPAL). Differences in cellular communication between groups can be analyzed by calculating the information flow, the sum of communication probability for each cell cluster predicted interaction, for each signaling pathway. Signaling network differences between groups can also be analyzed by performing manifold learning and classification based on functional similarity, or the similarity in sender/receiver cell types between two pathways. CellChat algorithm also allows analysis of specific cell type interactions within each group for each signaling pathway, and this can be visualized as a circular chord diagram. This analysis was used to determine the communication differences between B/My and T/My MPAL, particularly focusing on pathways that are estimated to have blast cells as the receiver or sender cells and might be key for disease progression.

### Stemness index

To further compare the relative levels of stemness of blast and normal cells, a stemness index was calculated for each cell as the first principal component value after a principal component analysis. The analysis was based on the expression values of 175 genes in the stemness index signature (Additional file 1: Table S3). This stemness index signature was identified by Palmer et al. to stratify hematopoietic malignant cells from normal and precursor cells [36].

### Survival analysis

Estimated survival probabilities for the T/My MPAL and B/My MPAL blasts biomarker sets were calculated using the survMisc v0.5.5 [37] and survival [38] v3.2–13 R packages. Survival analysis was performed on the TARGET-ALL-P3 dataset after obtaining the T-myeloid and B-myeloid MPAL samples [31], along with their overall survival in months and vital status information. For each gene in the T/My MPAL biomarker set (Additional file 1: Table S4), the T/My MPAL TARGET samples (Additional file 1: Table S5) were partitioned into high and low expression groups using the cutP method [37] from the survMisc package v0.5.5. The survival association of each gene was assessed by computing Kaplan–Meier survival curves and hazard ratio statistics via the *survfit* (with confidence type “log–log”) and *coxph* functions of the survival R package. This analysis allowed us to analyze the probability of survival over time and compare survival rates between groups with high and low expression of genes. A similar survival analysis was also performed for the B/My MPAL gene set in the B/My MPAL samples from the TARGET-ALL-P3 dataset.

## Results

### Single-cell RNA sequencing characterizes the heterogeneous nature of MPAL with identification of distinct profiles for immunophenotypic subtypes

Comprehensive single-cell transcriptome profiling was performed, after thawing viably frozen patient BM samples collected at the time of initial diagnosis (Dx), using the 10x Genomics platform. To robustly map the transcriptome landscape, we also included two MPAL samples from the ScPCA initiative [15]. In total, we performed analysis on nine pediatric MPAL samples (*n* = 8 at diagnosis, *n* = 1 at relapse) to understand underlying molecular mechanisms and explore potential therapeutic targets. To identify blast cells and study their transcriptome landscape, the data from healthy pediatric and young adult BM samples was included in the analysis [16]. In total, we analyzed the transcriptome profile of 67,024 cells (23,318 from five T/My MPAL, 17,258 from four B/My MPAL, and 26,448 from healthy BM samples). After quality control, filtering, and normalization, the unsupervised analysis identified 26 transcriptionally distinct clusters of cells (Additional file 2: Fig. S2). The annotation based on the individual samples depicted segregation in the single-cell profiles of MPAL subtypes from healthy BM samples (Fig. 1A). The cellular clusters were labeled based on the expression of canonical cell lineage-associated markers (Fig. 1B); these cell types include erythroblasts, monocytes, T-cells, B-cells, Pro-B, NK, and progenitor cells. The expression of these markers in individual sample’s blast cells is shown in Additional file 2: Fig. S3. The putative MPAL leukemic blast clusters depicted segregated clustering from the healthy normal cell clusters (Fig. 1C, non-blast cells lassoed). B/My MPAL blasts formed segregated clusters as compared to T/My MPAL, indicative of subtype heterogeneity (Fig. 1C). The gene expression of blast markers used in flow cytometry for immunophenotypic characterization is shown in Additional file 2: Fig. S4. On the other hand, immune cells from both MPAL subtypes clustered together with no subtype heterogeneity (Fig. 1C). Further individual sample analysis captured significant heterogeneity among blast cells from some patients (Additional file 2: Fig. S5). For example, T/My MPAL sample M2 and B/My MPAL sample M3 contained two and three distinct blast cell clusters on UMAP visualization respectively, correlating with the characterization on flow cytometry (Additional file 1: Table S2, and Fig. 1D). For the T/My MPAL M2 sample, there were two blast cell populations, which overexpressed myeloid (e.g., *S100A8, S100A9,* and *LYZ*) and T-lymphoid (e.g., *CD3D* and *CD3E*) lineage markers respectively (Additional file 1: Table S6A). Similarly, DEGs analysis between the two major blast populations in the B/My MPAL M3 sample (Additional file 1: Table S6B) showed over-expression of myeloid (e.g., *S100A8, S100A9,* and *LYZ*), and the B-lymphoid (e.g., *CD79B, CD79A,* and *MZB1*) lineage markers respectively. These results clearly depict the usefulness of single-cell profiling in highlighting the heterogeneity of blast cell populations, which is a unique attribute of MPAL cases. As anticipated, the profile of cancerous samples collected at the time of initial diagnosis or relapse was primarily composed of malignant blasts (Fig. 1E), with a limited enrichment of normal stromal and immune cell types. Available immune cell type breakdown from diagnostic flow cytometric characterization (Additional file 1: Table S2) correlated with the clusters seen in single-cell profiling. In contrast, the healthy BM samples consisted of major cell types from lymphoid and myeloid lineages including B-cells, T-cells, and natural killer (NK) cells (Fig. 1E).

The differential expression analysis between B/My or T/My MPAL blasts and healthy cells can provide insight into the molecular mechanisms underlying the development and progression of MPAL subtypes. The differential expression analysis based on fold change and *p*-value (Wilcoxon rank test, average log2FC > 0.25 and adjusted *p*-value < 0.05) identified 219 and 194 significant DEGs in B/My and T/My MPAL blast cells respectively. The top 20 genes based on average log2 fold change for MPAL subtypes were selected and plotted on a heatmap (Fig. 1F). B/My MPAL blasts depicted significant overexpression genes such as *STMN1* and *SOX4*, which have been previously associated with other hematological malignancies, such as AML and ALL [39, 40]. T/My MPAL blasts overexpressed genes such as *SPINK2* which has been associated with immune infiltration in AML [41], and *CD82* which has been identified as a driver of chemoresistance in AML [42]. Interestingly, B/My MPAL blasts up-regulated genes show high expression in healthy B/Pro-B-cells (Fig. 1F) indicating that these genes might play a role in the normal differentiation and maturation of B/Pro-B-cells. While T/My MPAL blasts overexpressed genes were observed to exhibit minimal or no expression in the normal T-cells derived from the young adult BM. We did not investigate the expression of these T/My MPAL blasts genes in precursor T-cells from the thymus, which represents a limitation of our study (Fig. 1F). To understand the dysregulations at Pathways and Gene ontology levels, we performed gene ontology (GO) enrichment analyses on the significantly overexpressed genes for the MPAL subtypes (Additional file 1: Table S7). The top significantly enriched (Benjamini–Hochberg adjusted *p*-value < 0.05) GO categories for B/My MPAL blasts are associated with activation and regulation of the immune response (Additional file 2: Fig. S6A). On the other hand, for T/My MPAL blasts overexpressed genes depicted significant enrichment in GO categories associated with cell signaling and negative regulation of myeloid and leukocyte differentiation (Additional file 2: Fig. S6B).

To further characterize the GO categories that are commonly dysregulated in T/My and B/My MPAL blast cells compared to healthy progenitor cells, we performed additional DEGs analysis comparing MPAL blast cells (*n* = 35,515) and healthy progenitor cells (*n* = 3902). The DEGs (Additional file 1: Table S8A, *n* = 147) were subsequently used to perform GO enrichment analysis to identify associated biological processes. The MPAL blasts overexpressed genes revealed significant associations with myeloid leukocyte migration, NF-kB transcription factor activity, and immune system processes (Fig. 1G). In addition, cellular communication analysis of B/My and T/My MPAL cells via the expression of ligands and receptors using the CellChat tool identified dysregulated signaling pathways associated with aberrant cellular communications. The macrophage migration inhibitory factor (MIF) signaling pathway was found to be enriched in both MPAL subtypes and was associated with signaling from blasts to immune cells including monocytes and B-cells (Fig. 1H). The gene for the ligand of the MIF signaling pathway (*MIF*) depicted significant up-regulation in both T/My and B/My MPAL blasts compared to healthy cells (Additional file 1: Table S8B). These results mapping the landscape of MPAL subtypes, highlight the inter- and intra-patient heterogeneity in blast cell profiles and similarities in the immune microenvironment landscape.

### Compared to other acute leukemias, the scRNAseq profiles of B/My MPAL show significant overlap with B-ALL and AML, while T/My MPAL shows overlap with AML and T-ALL

To assess the similarities and differences between MPAL and other acute leukemias, we performed comparative analyses among MPAL, AML, B-ALL, T-ALL, and healthy BM single-cell profiles. Single-cell transcriptome data for other leukemias (Additional file 1: Table S9) were obtained from the Pediatric Cancers Single-Cell Atlas [17, 18, 43] initiative of our lab and publicly available studies [16]. After uniform pre-processing, filtering, normalization, and batch correction, the gene expression profiles were visualized using UMAP (Fig. 2A). UMAP analysis showed that B/My MPAL blasts clustered mostly with B-ALL and AML (clusters 0, 4, and 6) due to presence of both myeloid and B-lymphoid features in the B/My MPAL blasts (Fig. 2A, Additional file 2: Fig. S7). On the other hand, T/My MPAL blasts profile overlapped with T-ALL and AML (clusters 0, 1, and 8) due to myeloid and T-lymphoid features in the T/My MPAL blast cells (Fig. 2A, Additional file 2: Fig. S7). Interestingly, immune cells clustered based on the cell lineage irrespective of leukemia type/subtype indicating minimal heterogeneity in the immune landscape; T and NK cells are located mostly in clusters in 3, 7, 13, and 24; B-cells in cluster 9; and monocytes in clusters 2, 5, and 16. To evaluate stemness levels, we also calculated the stemness index for the single-cell clusters based on the expression values of 175 genes in the stemness index signature identified by Palmer et al. (Additional file 1: Table S3) [36]. Higher stemness was observed for blast cells from different acute leukemias as compared to non-blast immune cells. The T/My MPAL blast cells showed the highest level of stemness compared to other blast populations, whereas the B/My MPAL blast cells stemness index was similar to B-ALL and AML blasts (Fig. 2B). A sample-wise DEG analysis was performed, to calculate sample-to-sample distances based on the DEGs. The analysis revealed that blast cells from B/My MPAL and B-ALL exhibit the most similar profiles, whereas blast cells from T/My MPAL are most similar to near ETP/ETP T-ALL samples (Additional file 2: Fig. S8).

To identify genes with significant overexpression in MPAL as compared to other leukemias and healthy controls, DEG analysis was performed based on the Wilcoxon rank test (FC > 0.25 and adjusted *p*-value < 0.05). Genes that are overexpressed in both MPAL subtypes blast cells compared to other leukemias and healthy controls include *CD81*, and *LMO2* (Fig. 2C). B/My MPAL blast cells overexpressed genes include *AUTS2*, *PAX5*, *MTRNR2L12*, and *HBEGF*. T/My MPAL blast cells overexpressed genes include *PTTG1IP*, *ESYT2*, *PTEN*, and *CALCOCO2*. To ensure markers are MPAL blast cell-specific, we performed additional filtering steps based on expression in the BM immune cells and hematopoietic stem cells (HSCs). The BM single-cell data of 391,505 immune cells and HSCs were obtained from the Human Cell Atlas (HCA) Initiative [44]. MPAL blast-specific genes were found to be minimally expressed in these HSCs and immune cells from HCA (Fig. 2D) in contrast to canonical immune cell markers such as *CD3D* for T-cells and *CD44* for HSCs, which had high expression in these cells. Interestingly, *CD81* had relatively high expression in the BM mesenchymal stem cells (MSCs) that constitute 0.06% (*n* = 239 cells) of the HCA dataset (Fig. 2D). Recent studies have shown compelling evidence that CD81 serves as a promising marker for identifying MSC-derived extracellular vehicles and intracellular communication [45]. The complete lists of MPAL blast markers with fold change and p-values from each step of the filtering process (Additional file 2: Fig. S1) are listed in Additional file 1: Table S4. The biomarker genes, *CD81* and *LMO2* which are expressed in both MPAL subtypes (Fig. 2D) had minimal or no expression in normal immune and stem cells, making these genes ideal potential candidates for targeting. In addition, the expression of the B/My and T/My MPAL biomarker genes was also assessed (Additional file 2: Fig. S9A, B) using the TARGET bulk RNA-seq MPAL, AML, and T-ALL datasets [31]. Some of the MPAL subtype blast markers showed higher expression in MPAL bulk RNA-seq data, and the rest showed noisier results. We do not expect the bulk-RNA-seq data to match our single-cell expression patterns completely as bulk RNA-seq data depicts the average expression profile of immune, stromal, and blast cells in a sample. The selected genes (*CHST11*, *GLS*, *PPP1R12A*) that are overexpressed in B/My and T/My MPAL blast cells in single-cell data, showed significantly higher expression (Wilcoxon rank test, *p*-value < 0.05) in MPAL versus T-ALL and AML bulk RNA-seq TARGET samples (Fig. 2E).

To further understand the potential pathway level dysregulation in MPAL blast genes, pathway enrichment analysis was performed on the MPAL subtypes (i.e., T/My, B/My MPAL) blasts significantly overexpressed genes (Additional file 1: Table S4). The pathways analysis depicted the significant enrichment (*p*-value < 0.05) of B/My MPAL overexpressed genes in multiple cell cycle, proliferation, and immune system-related pathways (Fig. 2F). This included activation of protein kinase C (PKC) via G-Protein coupled receptor, gastrin signaling in inflammatory response, angiotensin II receptor type 1 (AGTR1) signaling via p38, extracellular-signal-regulated kinase (ERK) and epidermal growth factor receptor (EGFR) signaling, and glucocorticoid receptor signaling, as well the role of IL8 typically seen in colorectal cancer (Fig. 2F). Similar analysis on T/My MPAL overexpressed genes depicted a significant association with cell cycle, cell adhesion, and immune response including tissue factor signaling, ERK1/2 signaling, IL6 signaling similar to that seen in prostate cancer, and *PTEN* pathways (Fig. 2G). The pathway enrichment analysis also depicted a significant (*p*-value < 0.05) effect on Sphingosine 1-phosphate receptor 2 (S1P2) signaling in both B/My and T/My MPAL (Fig. 2F, G). Interestingly, while the activation signaling of S1P2 was prominent in B/My MPAL, the inhibitory signaling was most enriched in T/My MPAL. This dissimilarity in S1P2 signaling could be attributed to the upregulation of *HBEGF* in B/My MPAL and *PTEN* in T/My MPAL (Fig. 2D). *HBEGF* is a downstream target of S1P2 signaling, resulting in the promotion of cell survival (Additional file 2: Fig. S10A); whereas *PTEN* is a direct target of S1P2 signaling, resulting in the inhibition of FAK1 and inhibition of cell migration (Additional file 2: Fig. S10B). The analysis on top B/My MPAL affected pathways depicted that *HBEGF* was involved in 10 out of 15 top enriched pathways (Fig. 2F), whereas *PTEN* was found to be involved in 12 out of the 15 top affected pathways in T/My MPAL (Fig. 2G). The complete lists of significantly enriched pathway maps along with p-values are listed in Additional file 1: Table S10. To further explore the role of MPAL blast cells overexpressed genes in cancer outcomes, we performed survival analysis using the Cox proportional hazards model in the TARGET-ALL-P3 dataset [31]. The high expression of the B/My MPAL blast marker *MTRNR212* had an association with poor survival (HR = 4.80, *p*-value = 0.059) in B/My MPAL samples. Whereas high expression of *PTEN*, a T/My MPAL blast marker gene depicted a significant association with poor survival (HR = 4.50, *p* = 0.040) in the T/My MPAL samples (Fig. 2H).

### T/My MPAL has higher similarity to ETP than with non-ETP T-ALL, but still displays unique myeloid characteristics

Given the emerging literature showing greater overlap between T/My MPAL and ETP-ALL, we decided to compare the single-cell transcriptomic profiles between the two groups as well as non-ETP T-ALL cases. Among the 11 T-ALL cases used in the analysis, four cases had ETP-ALL-like features (ETP-ALL, *n* = 1 and near ETP-ALL, *n* = 3), while the remaining seven were categorized as non-ETP T-ALL (Additional file 1: Table S11). Among the five T/My MPAL cases, three had ETP-like immunophenotypic blasts features on flow cytometry, aside from their myeloid lineage defining antigen expression, that resulted in a diagnosis of T/My MPAL (Additional file 1: Table S2).

To assess the scRNAseq similarities of T/My MPAL to T-ALL and ETP subtypes, we performed a focused analysis on these 16 samples (Fig. 3). The UMAP visualization depicted T/My MPAL, ETP and T-ALL formed distinct clusters with some overlaps among them (Fig. 3A). For the blast populations specifically, there exist clusters with cells from T/My MPAL and ETP/near-ETP (cluster 0) and T/My MPAL and non-ETP T-ALL (cluster 7), as well as a distinct myeloid cluster 3 (high expression of *S100A9*, *LYZ*, *CD14*) which contains mostly T/My MPAL blasts (Fig. 3B). Differential expression analysis was performed to determine the top overexpressed genes for near-ETP/ETP-ALL blast cells compared to non-ETP T-ALL and T/My MPAL blast cells compared to non-ETP T-ALL. This analysis found that there are 353 common genes overexpressed in near-ETP/ETP-ALL and T/My MPAL compared to T-ALL (Fig. 3C). An additional analysis was performed to compare the three groups, with *AES* and *CD3D* showing high expression in non-ETP T-ALL blast cells, and near-ETP/ETP blast cells over-expressing *CKLF* and *TASP1* (Fig. 3D). T/My MPAL blasts overexpress *VAMP8* and *SAT1* with high specificity compared to the other T-ALL blast cells (Fig. 3D). The DEG analysis identified 1,021 T/My MPAL, 639 near-ETP/ETP, and 831 non-ETP T-ALL overexpressed genes for blast cells (average log2FC > 0.25 and adjusted *p*-value < 0.05). GSEA was performed on these three sets of genes to determine which biological processes were significantly over-represented (adjusted *p*-value < 0.05) in each of the three subtypes’ blast cells. GSEA analysis identified T/My MPAL overexpressed gene enrichment in inflammatory and cell growth-related processes (Additional file 2: Fig. S11A). The non-ETP T-ALL blasts had high enrichment of cell differentiation processes (Additional file 2: Fig. S11B), and near-ETP/ETP overexpressed genes linked to transcription regulation and/or structural organization processes (Additional file 2: Fig. S11C) such as *PTEN*, *TNIK*, and *AUTS2* (Fig. 3E). Further stemness analysis interestingly revealed that near-ETP/ETP T-ALL blasts had the highest stemness index, followed by T/My MPAL and non-ETP T-ALL blasts. The T/My MPAL depict bimodal distribution of the stemness index ranging from high (similar to ETP) to low stemness (similar to T-ALL) indicating heterogeneity at the stemness level (Fig. 3F). To our knowledge, this is the first comparison of T/My MPAL and ETP-ALL on a single-cell transcriptomics level.

### Adult and pediatric MPAL have similar transcriptional landscapes

To determine the similarities and differences between adult and pediatric MPAL, we performed a comparative analysis by analyzing adult MPAL scRNAseq data (*n* = 6) from a publicly available study [14] (Additional file 1: Table S9). Adult MPAL data after normalization and quality control analysis was merged with pediatric MPAL samples and healthy BM. After performing clustering, annotation, and UMAP visualization inspection, we observed that the adult and pediatric samples predominantly clustered based on the MPAL subtypes rather than by age. This suggests that the MPAL subtypes are a more significant factor in determining the gene expression patterns than the age of the patients (Fig. 4A). In the UMAP, we observed that pediatric B/My MPAL samples (M1, M3, M5, M7) cluster together with the adult B/My MPAL sample (A4) in the lower left part of the plot. Specifically, these samples were distributed in clusters 2, 5, 9, 10, and 18. Similarly, the pediatric T/My MPAL samples (M2, M4, M6, SCPCS000230, SCPCS000220) cluster with the adult T/My MPAL samples (A1, A2, A3, A5, A5R) in the upper right portion of the UMAP in clusters 0, 1, 6, 8, and 27. This finding suggests a possible commonality in gene expression patterns between pediatric and adult MPAL subtypes. Furthermore, immune cells from adult and pediatric samples cluster together and are segregated based on cell type irrespective of age and MPAL subtypes (Fig. 4B).

To identify subtle potential age-specific MPAL blast markers, we performed differential expression analysis between adult and pediatric MPAL blasts (average log2FC > 0.25, adjusted *p*-value < 0.05). Heatmap with the top 20 markers (by average log2FC and percent expression) is shown in Fig. 4C. These genes might be linked to age instead of the malignant nature of the blasts. To further assess of stemness of blast cells from different pediatric and adult MPAL subtypes, we performed stemness analysis using a stem cell gene signature from literature [36]. In both MPAL subtypes, the adult patient blasts had higher stemness index values (Fig. 4D), indicating that pediatric MPAL blasts are more differentiated than adult MPAL blasts. Gene set enrichment analysis on pediatric and adult T/My MPAL blast cells provided insights into underlying biological mechanisms. Adult blast cells are significantly enriched (Wilcoxon rank-sum test, *p*-value < 0.001) with the inflammatory response and the KRAS up-regulated signaling pathway (Fig. 4E), while pediatric T/My MPAL blast cells are significantly enriched (*p*-value < 0.001) with TGF-β and PTEN pathways (Fig. 4F).

Overall, a comparison of adult and pediatric MPAL subtypes revealed a similar transcriptional landscape with subtle immune and inflammatory pathway level differences that might be due to the high mutational burden in adult MPAL and poorer outcomes.

### Diagnostic MPAL samples have transcriptomic differences that may help predict response to ALL induction therapy

**Fig. 5:**
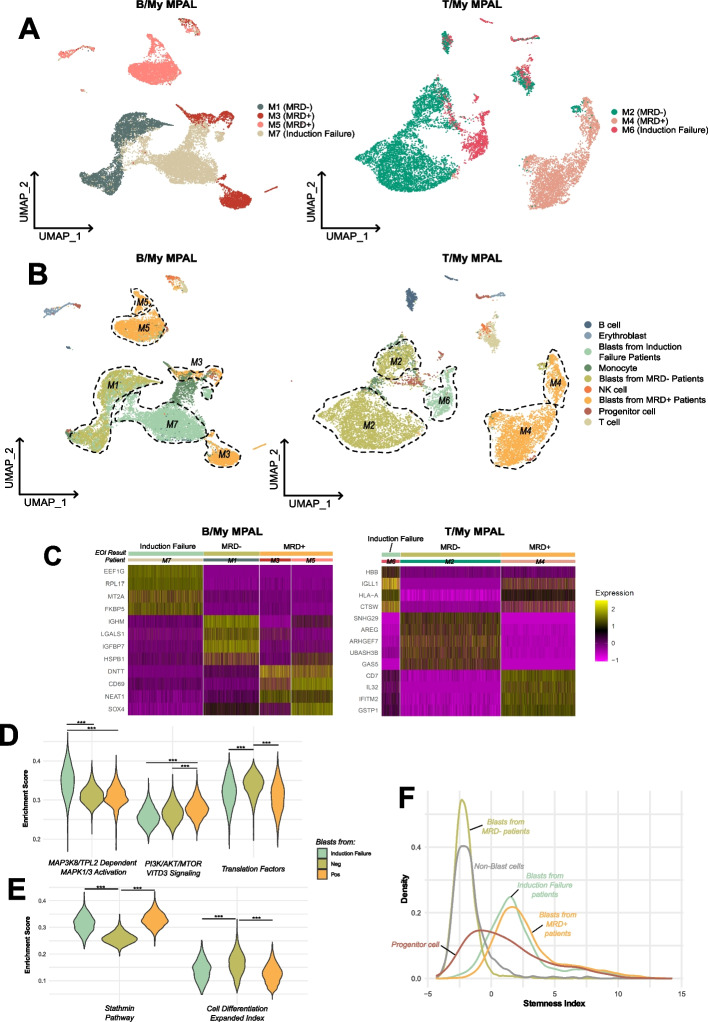
Comparison of the single-cell landscape of diagnosis MPAL samples based on induction outcomes. **A** UMAP plot of B/My (*n* = 17,258 cells) and T/My (*n* = 11,031 cells) MPAL patient cells colored by patient IDs, with the end of induction outcome (MRD + , MRD − , induction failure) information shown in the legend. **B** UMAP plots of B/My and T/My MPAL patient cells colored based on cell type including malignant blast (Blast cells from MRD + , MRD − , and induction failure patients) and normal cells (B-cells, T-cells, NK cells, Progenitor cells, monocytes, erythroblasts). **C** Heatmap showing top overexpressed genes in B/My MPAL and T/My MPAL blast cells from patients with different induction outcomes (induction failure, MRD + , and MRD −). The markers for the end of induction outcome group blasts were identified by comparing the target group’s blast cells with the other groups’ blast cells and filtered based on fold change, multiple test corrected *p*-value, and % expression (average log2FC > 0.25, adjusted *p* value < 0.05, pct. > 0.7). **D** Violin plots showing gene set enrichment values for different Biocarta and Reactome gene sets in B/My MPAL induction outcome blast groups calculated using single-sample gene set enrichment analysis. The significance between groups was calculated with Wilcoxon rank tests, with *p*-value < 0.001 represented with “***”. **E** Violin plots showing gene set with significantly different enrichments in T/My MPAL induction outcome groups. The significance between groups was calculated using Wilcoxon rank tests, with *p*-value < 0.001 represented with “***.” **F** Density plot showing stemness index distribution of the different cell types found in T/My MPAL samples. The stemness index was calculated as the first principal component value of each cell after performing principal component analysis with the expression of the genes in a stem cell gene set as the features (Additional file 1: Table S3). Populations of interest are shown in bolder lines and labeled

Given the growing consensus that MPAL patients should be initially treated with an ALL-directed induction regimen, we explored the association between transcriptome profiles at baseline and the end of induction (EOI) results following an ALL-based chemotherapy. Of the eight de novo MPAL cases, seven received initially an ALL-based induction regimen (Additional file 1: Table S1). These cases were partitioned into different groups based on their outcome status at the end of induction (i.e., MRD + , MRD − , and induction failure) (Table 1). Among the two MPAL induction failure patients, one had a poor initial response to ALL induction and was switched to AML therapy at day 13 (M7), hence was classified as an induction failure to ALL therapy. Given the distinct transcriptome profiles between the B/My and T/My MPAL subtypes, we chose to analyze the two groups separately. Among the B/My MPAL patients, two were MRD + (M3 and M5), one was MRD − (M1), and one had an induction failure, requiring change in therapy (M7). For the T/My MPAL patients, one had induction failure (M6), one was MRD + (M4), and one was MRD − (M2). As this analysis exclusively incorporates samples obtained at the time of diagnosis, the term “blasts from MRD + patients” refers to blast cells identified during the initial diagnosis of patients who were determined to have minimal residual disease (MRD +) at the end of induction (EOI). Clustering and UMAP embeddings based on induction results formed patient-specific clusters in B/My MPAL as well as T/My MPAL (Fig. 5A). One of the B/My MPAL MRD + (M5) patients formed a distant cluster indicating the highest transcriptional difference as compared to the cells from other MRD + , MRD − and induction failure patients. Additionally, MRD + patients also depicted significant heterogeneity in the blast cell profile as evident from multiple blast clusters of the same patient (Fig. 5B). The non-blast cells formed mostly overlapping clusters except monocytes (Fig. 5B). To identify baseline differences in the blasts based on induction status (MRD + , MRD − , induction failure), we performed differential expression analysis for B/My and T/My MPAL samples (Fig. 5C). B/My MPAL blast cells from induction failure patients showed high expression of *MT2A* and *FKBP5* genes that are associated with chemoresistance in osteosarcoma [46], solid cancers, and ALL [47]. The blast cells from the MRD − patients depicted the highest expression of *IGHM*, a gene associated with good prognosis in breast cancer [48], and *IGFBP7*, a marker of leukemia cell and chemosensitivity in AML [49]. Blast cells from B/My MPAL MRD + patients overexpressed genes such as *NEAT1* and *SOX4* that are associated with cancer development and pan-cancer poor outcome [50, 51]. For T/My MPAL the top marker genes for blast cells from induction failure and MRD + patients show significant overlap, whereas markers for blast cells from MRD − patients are more uniquely expressed. To further identify the key pathways over-represented in the three blast groups, we performed gene set enrichment analysis using canonical pathways gene sets from the MSigDB database [52]. Blast cells of B/My MPAL the induction failure patient depicted the highest enrichment (Wilcoxon ranked test, *p*-value < 0.001) of MAP3K8/TPL2 dependent MAPK1/3 activation (Fig. 5D). B/My MPAL blast cells from the MRD − patient had high enrichment of the translation factors gene set (Wilcoxon ranked test, *p*-value < 0.001) (Fig. 5D), whereas MRD + blast cells had high enrichment of the PI3K/AKT/mTOR VITD3 signaling pathway (Fig. 5D). Blast cells from T/My MPAL MRD − patient depicted significantly higher enrichment of the cell differentiation expanded index as compared to blast cells from MRD + and induction failure patients (Wilcoxon ranked test, *p*-value < 0.001) (Fig. 5E). Induction failure and MRD + patient blast cells showed higher enrichment of Stathmin pathway as compared to blast cells from MRD − patients (Fig. 5E). The comparative analysis of stemness index revealed a broader distribution of stemness in T/My MPAL blast cells T/My MPAL compared to ALL and AML patients, indicating a higher level of heterogeneity (Fig. 2B). Further assessment of variation in stemness [53] across future induction outcomes groups depicted a similar and higher stemness index of diagnosis blast cells from induction failure and MRD + patients as compared to the MRD − patients (Fig. 5F).

Although the small sample size limits definitive conclusions, our analysis reveals distinct baseline transcriptomic differences in blast cells based on outcomes. To establish the robustness of these findings, further validation is essential using larger sample sets in future studies.

### Diagnostic MPAL samples show distinct transcriptome profiles based on future clinical outcome

**Fig. 6:**
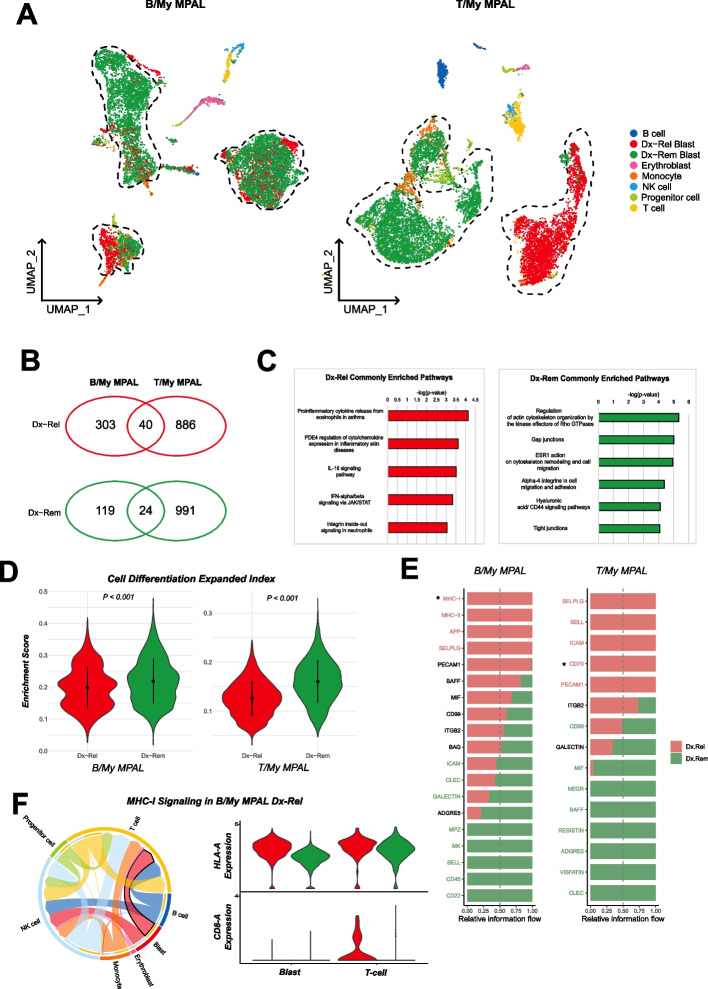
Exploratory analysis on T/My and B/My MPAL samples with relapse depicted differences in transcriptome profiles in comparison to samples with remission.** A** UMAP of Dx B/My MPAL cells (*n* = 10,591) annotated based on future clinical outcomes: relapse (Dx-Rel) or remission (Dx-Rem). The normal cells were annotated based on canonical markers (Fig. 1b). The B/My MPAL cells depict some overlapping Dx-Rem and Dx-Rel single-cell profiles on the unsupervised analysis. UMAP of Dx T/My MPAL cells (*n* = 11,031 cells) showing unique profile for Dx-Rel and Dx-Rem blasts with no overlap. **B** Venn diagram showing genes that are associated with MPAL remission or relapse in B/My and T/My MPAL. These genes were identified by comparing each subtype’s Dx-Rel and Dx-Rem blast cells profile and selecting significantly differentially expressed genes based on average log2FC > 0.25, and adjusted *p*-value < 0.05). The analysis identified 40 and 24 genes that are commonly upregulated in relapse and remission respectively at diagnosis. **C** Gene sets that are significantly associated with relapse (left) and remission (right) in MPAL at diagnosis. **D** Gene set enrichment analysis on the Dx-Rel and Dx-Rem blast cells in each MPAL subtype. The cell differentiation expanded index gene set depicted differential enrichment (Wilcoxon ranked test *p*-value < 0.05) between Dx-Rel and Dx-Rem blasts in both MPAL subtypes. **E** Cellular communication analysis based on ligand and receptor expression was performed to identify differences between remission and relapse outcomes at diagnosis. The pathways with significantly different information flow between remission and relapse (at Dx) B/My and T/My MPAL samples have been plotted as bar graphs. The information flow represents the sum of the communication probabilities of all cell types for the particular signaling pathway, and pathway names colored in pink and green representing enrichment for the Dx-Rel and Dx-Rem outcomes. **F** A chord diagrams for MHC-I signaling in Dx-Rel B/My MPAL cells. The left diagram shows the signaling between different cell types, from senders to receivers. The right diagram highlights the expression of ligand-receptor pairs estimated to interact between blasts and T-cells (green color represents Dx-Rem and red color represents Dx-Rel)

To determine if there are any transcriptomic features in the MPAL subtypes that are indicative of future relapse or remission status, we performed a comparison of samples collected at disease diagnosis based on outcomes. In the B/My MPAL subset, of the three patients who continued with ALL therapy post-induction, two patients achieved continuous complete remission (M1, M5) and one patient had a relapse (M3). In the T/My MPAL, two patients achieved continuous complete remission (M2, M6) and one patient relapsed after treatment (M4) with T-ALL chemotherapy regimens. The UMAP visualization based on outcomes (i.e., Remission = Dx-Rem, Relapse = Dx-Rel) depicted large transcriptome differences in T/My MPAL as compared to B/My MPAL (Fig. 6A). The differential expression analysis identified 343 and 926 genes that are significantly upregulated in relapse blast cells of B/My and T/My MPAL subtypes respectively (Fig. 6B). On the other hand, remission blast cells have 143 and 1015 significantly overexpressed genes in B/My and T/My MPAL subtypes respectively (Fig. 6B). The total number of differentially expressed genes between Dx-Rel and Dx-Rem blast cells was much higher for T/My MPAL (*n* = 1,941) as compared to B/My MPAL (*n* = 486), supporting our previous observation that the transcriptome landscape of T/My MPAL significantly different based on future outcome as compared to B/My MPAL. The comparative analysis of relapse-associated DEGs from both subtypes (i.e., B/My and T/My MPAL) identified 40 genes that are commonly associated with relapse outcomes in MPAL. A similar analysis identified 24 genes that are associated with remission outcomes in both MPAL subtypes (Fig. 6B). To understand the underlying biological mechanism associated with outcomes, we performed pathway enrichment analysis on 40 MPAL relapse- and 24 remission-associated genes using the MetaCore platform (Fig. 6C). Based on the commonly up-regulated markers in Fig. 6B, Dx-Rel blasts in MPAL depicted significant enrichment of genes associated with the cytokine and chemokine pathways, IL-16 pathway, and IFN-alpha signaling via JAK/STAT pathway (Fig. 6C) which are all involved in inflammation. In contrast, Dx-Rem blasts showed enrichment in cytoskeleton remodeling and cell adhesion/migration activity-related pathways. IFN-alpha has been shown to induce apoptosis and differentiation in AML cells, and there have been clinical trials for IFN-alpha targeting therapies in AML [54]. In MPAL Dx-Rel blasts, downstream targets of the IFN-alpha signaling via JAK/STAT are up-regulated (MX2, TNFSF10, IFI6, and IFITM1), which in turn have roles in the activation of apoptosis and the immune response (Additional file 2: Fig. S12). To further explore the differences in Dx-Rel and Dx-Rem blasts, we performed a gene set enrichment analysis. Dx-Rem blasts showed significantly higher enrichment (*p*-value < 0.05, Wilcoxon rank test) of the cell differentiation index gene set in both MPAL subtypes (Fig. 6D), indicating that the Dx-Rel blasts exhibit less differentiated features than Dx-Rem blast cells.

Cellular communication analysis was performed to highlight the differences in cellular communication and interactions between Dx-Rel and Dx-Rem samples to identify dysregulated signaling pathways. A comparison of the overall number of interactions and their strengths between Dx-Rel and Dx-Rem samples in B/My MPAL revealed most signaling was stronger in Dx-Rel samples, with some interactions involving blast and B-cells; whereas in T/My MPAL, Dx-Rem has more signaling overall but blasts have more signaling to T-cells in Dx-Rel. (Additional file 2: Fig. S13A, B). Further, we explored the information flow of the signaling pathways, which is calculated as the sum of communication probability among cell types of Dx-Rel and Dx-Rem samples (Fig. 6E). For both MPAL subtypes, SELPLG signaling was enriched in Dx-Rel samples, whereas CLEC signaling was enriched in Dx-Rem samples. In B/My MPAL specifically, MHC-I and MHC-II signaling was enriched in Dx-Rel samples, and the MHC-I pathway was explored further by plotting the specific cell types and ligand/receptor pairs that contribute to this communication probability (Fig. 6F). In B/My MPAL Dx-Rel, blasts, erythroblasts, monocytes, progenitor, and B-cells express MHC-I molecules and are estimated to be communicating with T and NK cells. When blasts and T-cells ligand-receptor pair interactions are plotted, the ligand (HLA-A) is expressed highly by Dx-Rel blasts, and the CD8A receptor is expressed exclusively in Dx-Rel T-cells (Fig. 6F) compared to Dx-Rem. In the T/My MPAL Dx-Rel cells, CD70 signaling was enriched and blast cells were predicted to be interacting with T-cells in the Dx-Rel sample via the CD70-CD27 ligand-receptor interaction (Additional file 2: Fig. S13C).

## Discussion

The emergence and optimization of single-cell profiling as a powerful tool to characterize the tumor microenvironment has revealed the heterogeneity of cancers, particularly different leukemia subtypes. Most MPAL biology studies to date, however, have focused on genetics and bulk RNA profiling that measures an average signal from the amalgam of blast and immune microenvironment cells in the bone marrow, failing to address blast cell heterogeneity, blast and immune cell interactions, and the role of the immune microenvironment in clinical outcome. There has only been one study published utilizing a single-cell approach to analyze this rare leukemia. In this study, Granja et al. analyzed samples from five adult MPAL patients and compared their findings to controls for normal hematopoiesis [14]. They demonstrated that despite widespread epigenetic heterogeneity within the patient cohort, common malignant signatures across patients were observed. Pediatric MPAL research is critical because in other leukemias like AML significant differences have been demonstrated between adult and pediatric leukemia microenvironments [55–57]. Also, the analysis by Granja et al. only included one patient with B/My MPAL; hence they did not perform a comparative analysis between the different B/My MPAL subtypes [14]. Therefore, besides being the first study to characterize the single-cell tumor landscape in pediatric MPAL patients, our study is also the first study to compare single-cell expression profiles between the two major MPAL subtypes.

Comparative analysis of gene expression patterns showed that the two MPAL subtypes, B/My and T/My MPAL, had distinct single-cell transcriptomic profiles. The B/My MPAL cases showed significant clustering overlap with B-ALL and AML. The T/My MPAL cases showed more overlap with T-ALL and AML. Individual differences between subtype populations were seen, likely due to differences in genomic drivers for individual cases. Previous large bulk genomic studies in pediatric MPAL have shown that B/My MPAL and T/My MPAL had distinct genetic profiles based on transcriptome and whole-genome sequencing [8]. B/My MPAL were shown to commonly have ZNF384 fusions, while T/My MPAL was associated with WT1 and FLT3 alterations. Available clinical grade cytogenetic and molecular profiling in our study did not reveal any ZNF384 fusions in our B/My MPAL samples, but alterations in FLT3 were seen in one T/My MPAL sample. Interestingly, ZNF384 fusions are also commonly seen in B-ALL suggesting overlapping biology between the two entities. Based on prior MPAL diagnostic criteria, often the switch in classification from B-ALL and B/My MPAL resulted from isolated MPO expression seen on otherwise typical appearing B-ALL cells. Previous work by our group showed that B/My MPAL cases with isolated MPO essentially clinically behaved like typical B-ALL, with an excellent response to ALL therapy [3]. The most recent WHO diagnostic criteria for MPAL published in 2022 [5] now accounts for the intensity of the lineage-defining marker (e.g., MPO), requiring it to be at least 50% when compared to expression in the most similar population (e.g., neutrophils for myeloid lineage). All MPAL samples in our study met these newly defined stricter criteria. Interestingly, among the four B/My MPAL samples analyzed in our cohort, one had a *BCR-ABL* translocation and one had a *KMT2A*-rearrangement, thus accounting for two major B/My MPAL categories defined by genetic abnormalities in the new criteria. Studies have also shown that T/My MPAL have a similar mutational profile to ETP-ALL [58], and more recent literature has shown that a subset of these cases, both in T/My MPAL and ETP-ALL, are associated with *BCL11B* (14q32) rearrangements. One of our samples had a t(7;14) (q21;q32) abnormality. Comparison of T/My MPAL single-cell profiles with ETP and non-ETP T-ALL cases showed greater overlap of T/My MPAL with ETP-ALL as one may expect; however, certain T/My MPAL samples had distinct non-overlapping clusters with greater myeloid antigen expression (*LYZ*, *CD74*) confirming this indeed is a separate entity. Overall, these findings suggest that the two MPAL immunophenotypic subtypes should be considered distinct entities and may have implications for differing treatment regimens, such as using more T-ALL-specific drugs like nelarabine for T/My MPAL. While there is no clear consensus as to how to treat MPAL patients, more recent literature has suggested utilizing an ALL-directed therapy approach first compared to AML therapy. Our results do support this as an initial approach given the overlap of MPAL with their corresponding ALL subtypes, but there remains wide heterogeneity among individual cases with some cases showing a much greater overlap with AML. As we continue to refine and improve our MPAL diagnostic criteria, thereby excluding potential ALL cases that previously met a weaker MPAL definition, it will be interesting to see if the more recent literature suggesting improved response to ALL therapy holds true in the future. A prospective clinical trial, which is a first for this rare disease, is now utilizing this treatment approach for de novo pediatric MPAL (NCT03959085).

Specific genes that were overexpressed in the blasts of both MPAL subtypes compared to other acute leukemias included *CD81* and *LMO2*. CD81, which is a member of transmembrane 4 superfamily, has been associated with a poor prognosis in AML [59] and is also a known marker in B-ALL [60, 61]. Interestingly, preclinical models have shown that *CD81* knockout promotes chemosensitivity and disrupts in vivo homing and engraftment in ALL [62]. LIM-domain only 2 (*LMO2*) is overexpressed in T-ALL and plays a critical role in the regulation of hematopoietic cell development as well as in DNA repair [63]. Over-expression of LMO2 in diffuse large B-cell lymphoma was shown to result in the accumulation of DNA double-stranded breaks, contributing to tumor cell genetic instability and chemosensitivity to PARP inhibitors [64]. Subtype analysis showed that B/My MPAL blast-specific genes included *AUTS2*, M*EF2A*, *RB1CC1*, and *HBEGF*, whereas T/My MPAL blasts overexpressed *PTEN*, *ESYT2*, *KMT2C*, and *PTGG1IP*. Heparin-binding epidermal growth factor-like growth factor (*HBEGF*) has been implicated in several cancers including leukemias as an inducer of tumor growth [65–67], and has previously been targeted in various cancers with small molecule inhibitors [67–69]. *PTEN* is typically considered a tumor suppressor gene, with inactivation leading to the development of T-ALL and AML [70, 71], thus the significance of *PTEN* overexpression in T/My blasts needs further exploration. Pathway enrichment analysis showed that HBEGF was involved in several enriched pathways in B/My MPAL, whereas PTEN was involved in the top affected pathways in T/My MPAL. Interestingly, both MPAL subtype blasts showed upregulated sphingosine 1-phosphate receptor 2 (S1P2) signaling, but with opposite downstream effects. B/My MPAL blasts had increased S1P2 receptor activation resulting in the promotion of cell survival and cell migration, while T/My MPAL blasts had elevated S1P2 receptor inhibitory signaling resulting in inhibition of cell migration and cell proliferation (Additional file 2: Fig. S10). S1P2 receptor has been researched as an inhibitor to invasion and metastasis in tumor cells, and up-regulation of S1P2 receptor inhibitory signaling has been proposed as a promising anti-cancer therapy [72].

Comparison of our pediatric MPAL samples with adult MPAL data from the Granja et al. study [14] showed that MPAL samples cluster together more by subtype than age, further indicating the transcriptomic differences between the subtypes. While there are few differences between adult and pediatric samples in clustering locations, differentially expressed genes were found between the two age groups, with adult MPAL patients having higher expression of genes related to HSC regulation and function in the bone marrow, *CD44* and *U2AF1* [73, 74]. In addition, adult MPAL subtype blasts had higher levels of the stemness than pediatric subtypes, indicating that adult MPAL blasts are less differentiated than pediatric MPAL blasts.

Finally, we concluded our paper with an exploratory analysis looking at the association of baseline MPAL single-cell profile and response to ALL-directed therapy. More recent literature has shown that pediatric MPAL patients with MRD at EOI have significantly poorer outcomes [4, 11]. In the multi-national iBFM-AMBI2012 study, Hrusak et al. showed that patients with MRD at EOI had a significantly worse event-free survival (EFS) and overall survival (OS), despite their analysis being complicated by the inclusion of a myriad of treatment regimens [4]. Oberley et al. similarly also showed that MRD positivity was highly predictive of relapse and death. Based on these findings, we first performed a comparative analysis by grouping our diagnostic samples according to their response to ALL induction therapy MRD: − , MRD + , or induction failure. Interestingly, we did identify differences in gene expression profiles based on induction response, with differences seen in stemness, cell cycle patterns, and specific pathway enrichments. However, given the limited sample size, a much larger sample size analysis will be needed to validate these interesting findings. We also performed a comparative analysis on overall remission vs relapse status and identified similar transcriptomic differences. Thus, while our findings are indeed preliminary and need to be validated with larger sample sets, it suggests that unique transcriptome profiles at diagnosis can be associated with response to ALL induction and overall therapy. Identifying these specific gene expression profiles associated with induction response in the separate MPAL subtypes would allow for better risk stratification and more tailored therapy for MPAL in future prospective clinical trials.

## Conclusions

Our data provides the initial framework of the single-cell landscape of pediatric MPAL. Comparison between B/My and T/My MPAL subtypes showed distinct transcriptome patterns and identified unique gene signatures and pathways specifically enriched in each subtype. T/My MPAL was shown to have more overlap with ETP-ALL cases, and a comparison between adult and pediatric MPAL blast signatures revealed only minor differences. We also saw differences in baseline transcriptome signature depending on the eventual response to ALL induction therapy, but a larger sample size is needed to validate these findings. Our future goal is to perform a more detailed integrative single-cell multiomic analysis on a larger set to pediatric MPAL samples, and to utilize newly identified signatures and targets for the development of novel diagnostics and therapies in the future.

### Supplementary Information

**Additional file 1:** **Table S1.** Detailed patient characteristics for pediatric MPAL samples analyzed in our study. For each MPAL sample analyzed, the sample ID, data source, diagnosis subtype, initial WBC (white blood cell count), PB (peripheral blood) blast percentage, BM (bone marrow) blast percentage, timepoint, induction regimen, EOI (end of induction) MRD (minimal residual disease) status, BM blast percentage at EOI, post induction therapy course, whether the patient relapsed or had refractory disease, whether the patient was alive or deceased at last f/u (follow up), and the time to death or last follow up (in days). **Table S2.** Flow cytometry characteristics of MPAL samples. The information includes the sample ID, diagnosis subtype, whether multiple blast populations were found, whether there was ETP-ALL or near ETP-ALL features, a description of the peripheral blood flow analysis, a description of the bone marrow flow analysis, the bone marrow blast percentage by morphology, the distribution of immune cells in bone marrow based on flow, and the percentage of immune cell subsets in the single-cell dataset (percentage of entire sample). **Table S3.** Stemness signature that was used for stem cell enrichment analysis. The stem cell enriched signature of 189 genes associated with high expression in stem cells across multiple tissue and disease types was obtained from Palmer et al. [34] study. **Table S4.** MPAL subtype blast biomarker genes. The table list the gene after each filtering step described in Additional file 2: Fig. S1. **Table S5.** Sample information for bulk RNA-seq TARGET samples. The sample ID, acute leukemia subtype, vital status, overall survival, and event-free survival for each sample were retrieved from the TARGET web portal (https://www.cancer.gov/ccg/research/genome-sequencing/target). **Table S6.** Top 40 differentially expressed genes (DEGs) between blast populations within T/My sample M2 and B/My sample M3. The top differentially expressed genes between blast subpopulations in B/My sample M3 (M3-My and M3-B) and in T/My sample M2 (M2-My and M2-T). DEGs analysis was performed for sample M3, comparing sample M3’s cells in clusters 0 versus 4, and for sample M2, comparing sample M2’s cells in clusters 4 and 7 versus 5. Please refer to Additional file 2: Fig. S2 for cluster information. Genes with adjusted *p*-value<0.05 and avg. log2FC>0.25 were considered significantly differently expressed, and the top 40 over-expressed genes were identified based on the highest avg. log2FC. **Table S7.** Significantly overexpressed genes in MPAL subtype blasts compared to healthy cells. The overexpressed genes were selected based on fold change (avg. log2FC > 0.25) and multiple test corrected *p*-value (Bonferroni correction, adjusted *p*-value<0.05). **Table S8.** Commonly overexpressed genes between MPAL subtypes. A) The significantly overexpressed genes (*n*=146, avg. log2FC > 0.25, adjusted *p*-value<0.05) in MPAL blast cells compared to healthy progenitor cells. B) The commonly significantly overexpressed genes (*n* = 75) between B/My and T/My MPAL, when compared to healthy BM cells (Additional file 1: Table S7). **Table S9.** Sample information for comparison of MPAL with other pediatric acute and adult MPAL. The sample ID, acute leukemia subtype, data source, and cell count for each sample are listed that were used for analysis shown in Figs. 2, 3, and 4. **Table S10.** Enriched pathway for B/My MPAL and T/My MPAL blast biomarker gene sets. The final MPAL blast biomarker genes (Additional file 1: Table S4) were used for pathway enrichment analysis using the MetaCore platform that contains functions, pathways, and network models derived by systematically exploring peer-reviewed scientific literature and public databases. The pathways with *p*-value<0.05 were considered significant. **Table S11.** Flow cytometry characteristics of T-ALL samples. The information includes the sample ID, flow cytometry characterization, along with non-ETP, the ETP-,ALL, and near ETP-ALL classification.

**Additional file 2:** **Figure S1.** A schematic overview describing T/My and B/My MPAL biomarker identification. Candidate markers were identified using the Seurat FindMarkers function, comparing MPAL subtype blast cell profile versus ALL, AML blast cells, and healthy immune cells (log2FC>0.25, adjusted *p*-value < 0.05, and percent expressed>0.5). The candidate differentially expressed genes were filtered using the Human Cell Atlas (HCA) healthy bone marrow dataset to identify genes with low expression in healthy data (average expression less than 0.5 in all clusters of HCA immune cells and hematopoietic stem cells (HSCs)). **Figure S2.** UMAP clusters of Mixed Phenotype Acute Leukemia and healthy cells. An unsupervised, KNN graph-based clustering method was applied to generate 26 distinct clusters of cells for the mixed phenotype acute leukemia (MPAL) and healthy bone marrow samples. These clusters are shown on dimensions UMAP_1 and UMAP_2 using the uniform manifold approximation and projection (UMAP) dimensionality reduction method. **Figure S3.** Dot plot with immune cell canonical marker expression for the MPAL and healthy cells. The y-axis shows the different MPAL blast clusters, and the x-axis shows the common immune cell markers. The dot color represents the level of expression, and the larger the dot the more of the cells in that cluster expressed the gene. **Figure S4.** Single-cell RNA expression of markers used for clinical diagnosis with flow cytometry. The violin plots show the log normalized, batch corrected expression values for each flow cytometry marker (with alternative gene names) in the MPAL samples’ blast cells. **Figure S5.** Feature plots highlighting cells for each MPAL sample. The cell locations of the four B/Myeloid MPAL samples (M1, M3, M5, M7) and the five T/Myeloid MPAL samples (M2, M4, M6, SCPCS000220, SCPCS000230) are highlighted in separate UMAP plots. **Figure S6.** Enriched GO Biological Processes in the genes significantly overexpressed (avg.log2FC>0.25, *p*-value<0.05) in MPAL subtypes. A) Top 20 enriched gene sets in the B/My MPAL blast cells as compared to healthy cells. B) Top 20 enriched gene sets in the T/My MPAL blast cells as compared to healthy cells. The size of the dot represents the number of genes that belong to a gene-set, the Gene Ratio represents the size of the overlap between blast cells overexpressed query genes and a given gene-set, and the color of the dots represent the significance of association based on “BH” adjusted *p*-values. **Figure S7.** UMAP single-cell clusters of Acute Leukemias and healthy bone marrow cells. A) An unsupervised, KNN graph-based clustering method was applied to generate 33 distinct clusters of acute leukemias and healthy bone marrow samples. B) Dot plot showing the percent of each blast cell type of acute leukemia contributing toward cell clusters. The clusters of interest are shown in the grey boxes. The dot size and color represent the proportional size of the cluster (number of cells in cluster / total cells in object) and percent contribution of blast cell type toward each cluster respectively. The red and blue colors represent th e high and low contribution of cell types in the clusters respectively. **Figure S8.** Average sample to sample distances between subtypes based on common over-expressed genes. The over-expressed genes (average log2FC>0.25 and adjusted *p*-value<0.05) were found when comparing sample blast cells to each other. To assess their differences, the Jaccard distance (1 – size of intersection divided by size of union) was calculated for each pair of samples. The average distance for the sample pairs between two subtypes was calculated and plotted on a heatmap. The blue color represents subtypes that are more different and pink represents subtypes that are more similar based on their common blast over-expressed genes between sample pairs. **Figure S9.** Expression of MPAL blast biomarker genes in the bulk RNA-Seq data. A) Scaled log2(expression+1) for each of the B/My MPAL blast biomarkers. B) Scaled log2(expression+1) for each of the T/My MPAL blast biomarkers. Transcripts per million (TPM) values for bulk RNA – Seq data were downloaded from the TARGET initiative portal (https://www.cancer.gov/ccg/research/genome-sequencing/target). The significance between groups was calculated using Wilcoxon rank tests, with “***” representing *p*-value<0.001, “**” representing *p*-value<0.01, “*” representing *p *value<0.05, and “ns” representing *p*>0.05. **Figure S10.** Pathway enrichment of significantly over-expressed genes in MPAL subtypes blast cells. A) S1P2 receptor activation signaling pathway, with significant upregulation of HBEGF, MEK1/2 (MAP2K2), and MLCP (PPP1R12A) in the B/My MPAL biomarker set and marked with the red square. B) S1P2 receptor inhibitory signaling pathway, with PTEN, MLCP (PPP1R12A), MEK1/2 (MAP2K2), and Alpha-actin (ACTN4) significant upregulation in the T/My MPAL biomarker set and marked with the red square. **Figure S11.** Top unique enriched Gene Ontology gene sets for T/My MPAL (A), non-ETP T-ALL (B), and near-ETP/ETP-ALL (C) blast marker sets. The gene ontology analysis was performed using clusterProfiler and Biological Process GO categories with Benjamini–Hochberg *P* value < 0.05 are considered significant. **Figure S12.** Detailed view of IFN-alpha/beta signaling via JAK/STAT pathway that was significantly affected (*p*-value<0.05) in the commonly over-expressed genes for Dx-Rel (future relapse) as compared to Dx-Rem (future remission) blast cells in both MPAL subtypes. The pathway enrichment analysis was performed using the MetaCore platform. The genes of the signaling pathway that were significantly up-regulated in the Dx-Rel blast cells are highlighted with a red dotted box. **Figure S13.** Cellular communication circle plots for Dx-Rel and Dx-Rem cells in MPAL subtypes. A) The differential number of interactions between Dx-Rel and Dx-Rem samples for each subtype. The cellular communication was estimated based on the ligand and receptor expression between interacting cell types and shown with arrows. The thickness of the arrow represents the relative number of interactions among cell types. B) The differential interaction strength between Dx-Rel and Dx-Rem samples. Each arrow represents the relative strength of interactions among cell types. The arrows are colored in red and blue depending on the higher number or strength interactions in Dx-Rel or Dx-Rem samples respectively. C) CD70 signaling in T/My MPAL Dx-Rel cells. The chord diagram shows the sender and receiver cell type, along with the ligand (CD70) and receptor (CD27).

## Data Availability

The single-cell count matrices for the MPAL samples processed in our lab (M1-M7) are publicly available on GEO (GSE236351), at https://www.ncbi.nlm.nih.gov/geo/query/acc.cgi?acc=GSE236351 [75]. The two T/My MPAL and two healthy BM samples count matrices obtained from the ScPCA (SCPCP000007) are available at https://scpca.alexslemonade.org/projects/SCPCP000007 [15]. The four young adult healthy, eight AML, and seven B-ALL samples’ count matrices obtained from GEO (GSE154109) are available at https://www.ncbi.nlm.nih.gov/geo/query/acc.cgi?acc=GSE154109 [16, 23]. Three additional pediatric healthy sample count matrices were obtained from GEO (GSE132509), at https://www.ncbi.nlm.nih.gov/geo/query/acc.cgi?acc=GSE132509 [19, 24]. We retrieved eleven T-ALL (GSE227122, https://www.ncbi.nlm.nih.gov/geo/query/acc.cgi?acc=GSE227122) and fifteen AML (GSE235923, https://www.ncbi.nlm.nih.gov/geo/query/acc.cgi?acc=GSE235923) count matrices from the previously published studies from our lab [17, 18, 21, 22]. The adult MPAL sample count matrices were retrieved from GEO (GSE139369), and are available at https://www.ncbi.nlm.nih.gov/geo/query/acc.cgi?acc=GSE139369 [14, 25]. In addition, our lab has developed an interactive web tool to access and analyze all pediatric samples included in this study, at https://bhasinlab.bmi.emory.edu/PediatricSCAtlas/.
