# Supplementary material for: Single-cell RNA sequencing distinctly characterizes the wide heterogeneity in pediatric mixed phenotype acute leukemia
Source: Genome Med. 2023 Oct 16;15:83. doi: 10.1186/s13073-023-01241-z (PMC10577904; doi:10.1186/s13073-023-01241-z)
Supplement: Supplementary file 2 — Additional file 2: Figure S1. A schematic overview describing T/My and B/My MPAL biomarker identification. Candidate markers were identified using the Seurat FindMarkers function, comparing MPAL subtype blast cell profile versus ALL, AML blast cells, and healthy immune cells (log2FC>0.25, adjusted p-value < 0.05, and percent expressed>0.5). The candidate differentially expressed genes were filtered using the Human Cell Atlas (HCA) healthy bone marrow dataset to identify genes with low expression in healthy data (average expression less than 0.5 in all clusters of HCA immune cells and hematopoietic stem cells (HSCs)). Figure S2. UMAP clusters of Mixed Phenotype Acute Leukemia and healthy cells. An unsupervised, KNN graph-based clustering method was applied to generate 26 distinct clusters of cells for the mixed phenotype acute leukemia (MPAL) and healthy bone marrow samples. These clusters are shown on dimensions UMAP_1 and UMAP_2 using the uniform manifold approximation and projection (UMAP) dimensionality reduction method. Figure S3. Dot plot with immune cell canonical marker expression for the MPAL and healthy cells. The y-axis shows the different MPAL blast clusters, and the x-axis shows the common immune cell markers. The dot color represents the level of expression, and the larger the dot the more of the cells in that cluster expressed the gene. Figure S4. Single-cell RNA expression of markers used for clinical diagnosis with flow cytometry. The violin plots show the log normalized, batch corrected expression values for each flow cytometry marker (with alternative gene names) in the MPAL samples’ blast cells. Figure S5. Feature plots highlighting cells for each MPAL sample. The cell locations of the four B/Myeloid MPAL samples (M1, M3, M5, M7) and the five T/Myeloid MPAL samples (M2, M4, M6, SCPCS000220, SCPCS000230) are highlighted in separate UMAP plots. Figure S6. Enriched GO Biological Processes in the genes significantly overexpressed (avg.log2FC>0.25, p-v [file 13073_2023_1241_MOESM2_ESM.pdf]

Fig S1

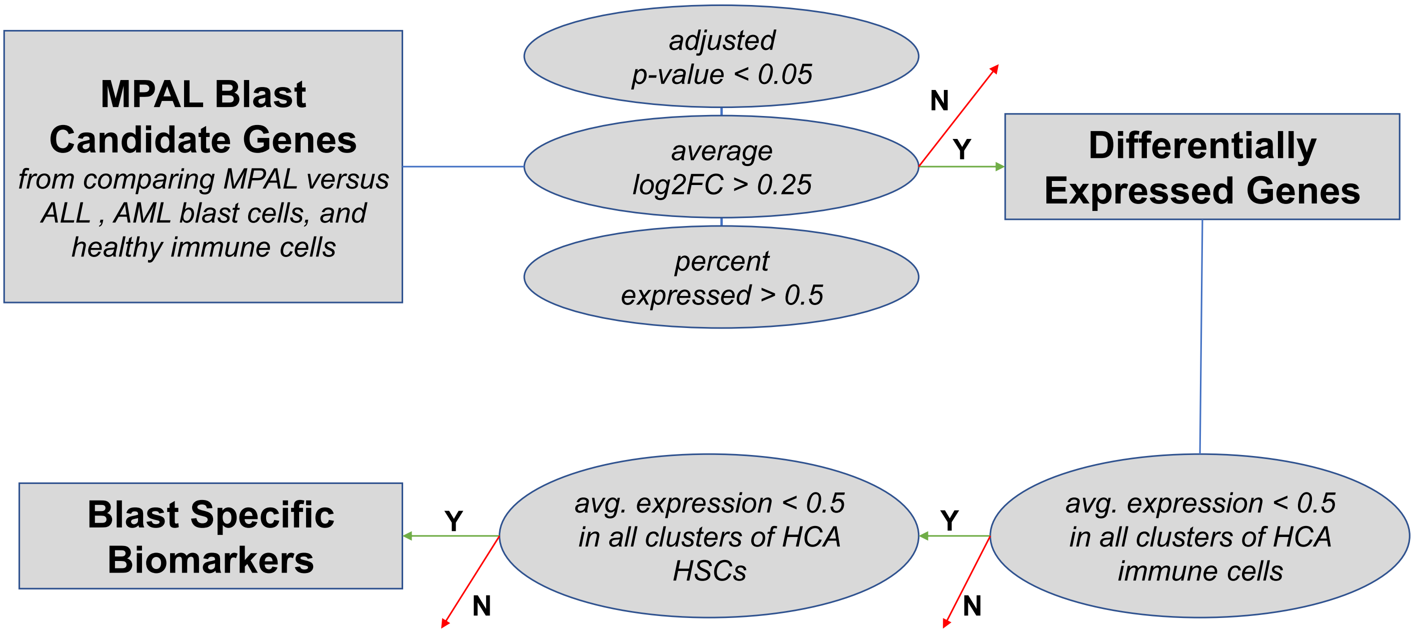

Figure S1. A schematic overview describing T/My and B/My MPAL biomarker identification. Candidate markers were identified using the Seurat FindMarkers function, comparing MPAL subtype blast cell profile versus ALL, AML blast cells, and healthy immune cells (log2FC>0.25, adjusted p-value < 0.05, and percent expressed>0.5). The candidate differentially expressed genes were filtered using the Human Cell Atlas (HCA) healthy bone marrow dataset to identify genes with low expression in healthy data (average expression less than 0.5 in all clusters of HCA immune cells and hematopoietic stem cells (HSCs)).

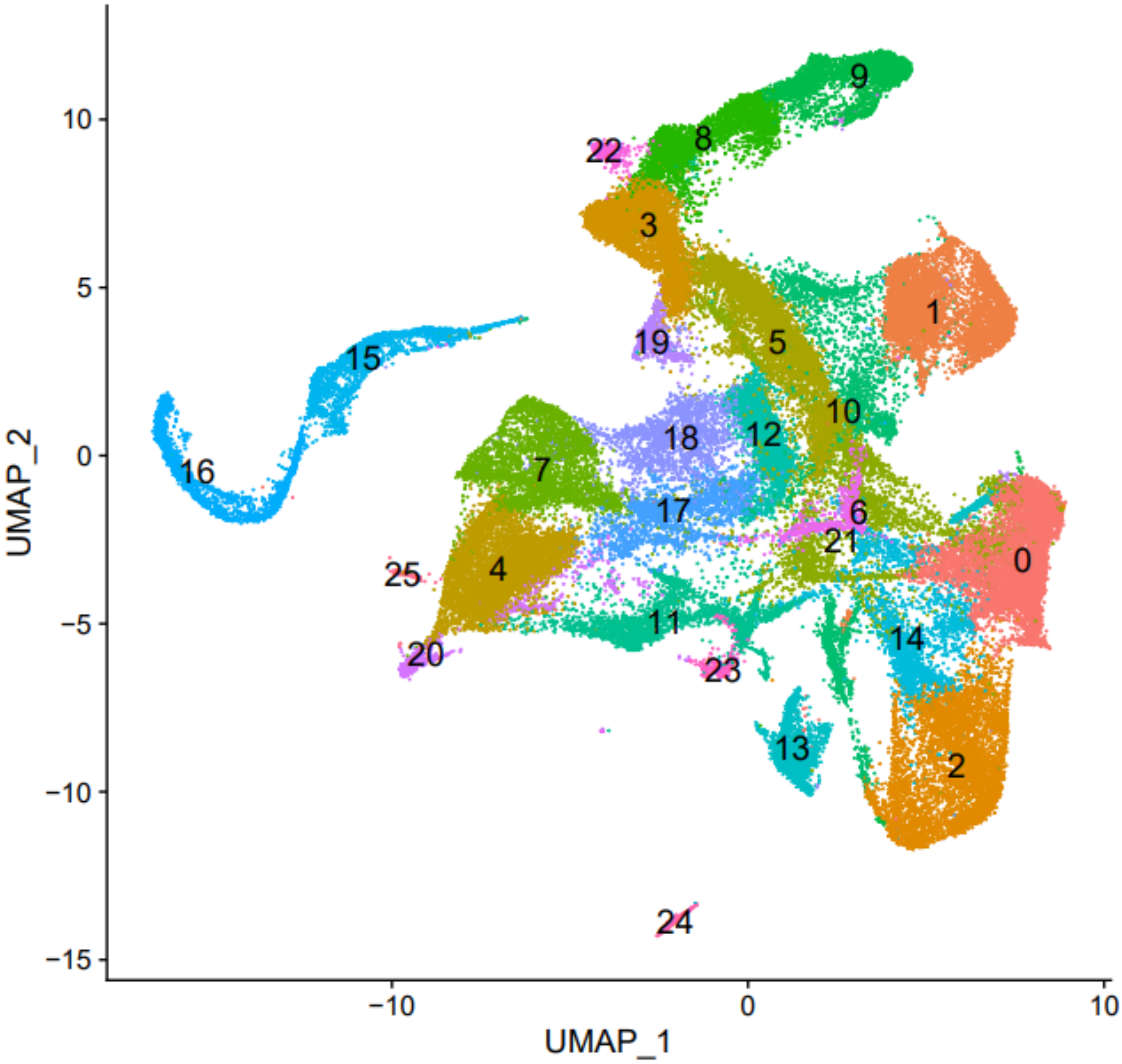

Figure S2. UMAP clusters of Mixed Phenotype Acute Leukemia and healthy cells. An unsupervised, KNN graph-based clustering method was applied to generate 26 distinct clusters of cells for the mixed phenotype acute leukemia (MPAL) and healthy bone marrow samples. These clusters are shown on dimensions UMAP_1 and UMAP_2 using the uniform manifold approximation and projection (UMAP) dimensionality reduction method.

Fig S3

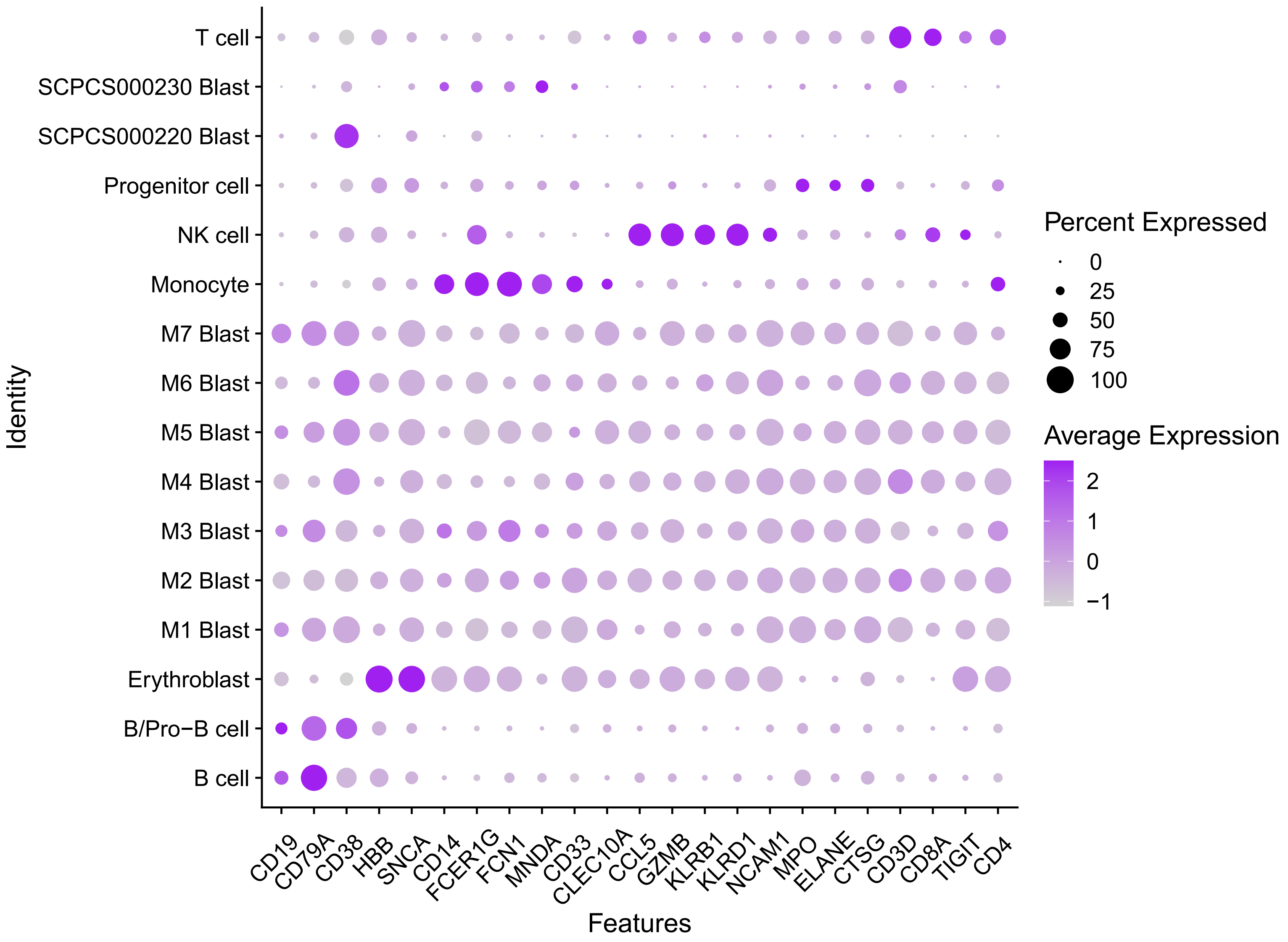

Figure S3. Dot plot with immune cell canonical marker expression for the MPAL and healthy cells. The y-axis shows the different MPAL blast clusters, and the x-axis shows the common immune cell markers. The dot color represents the level of expression, and the larger the dot the more of the cells in that cluster expressed the gene.

Fig S4

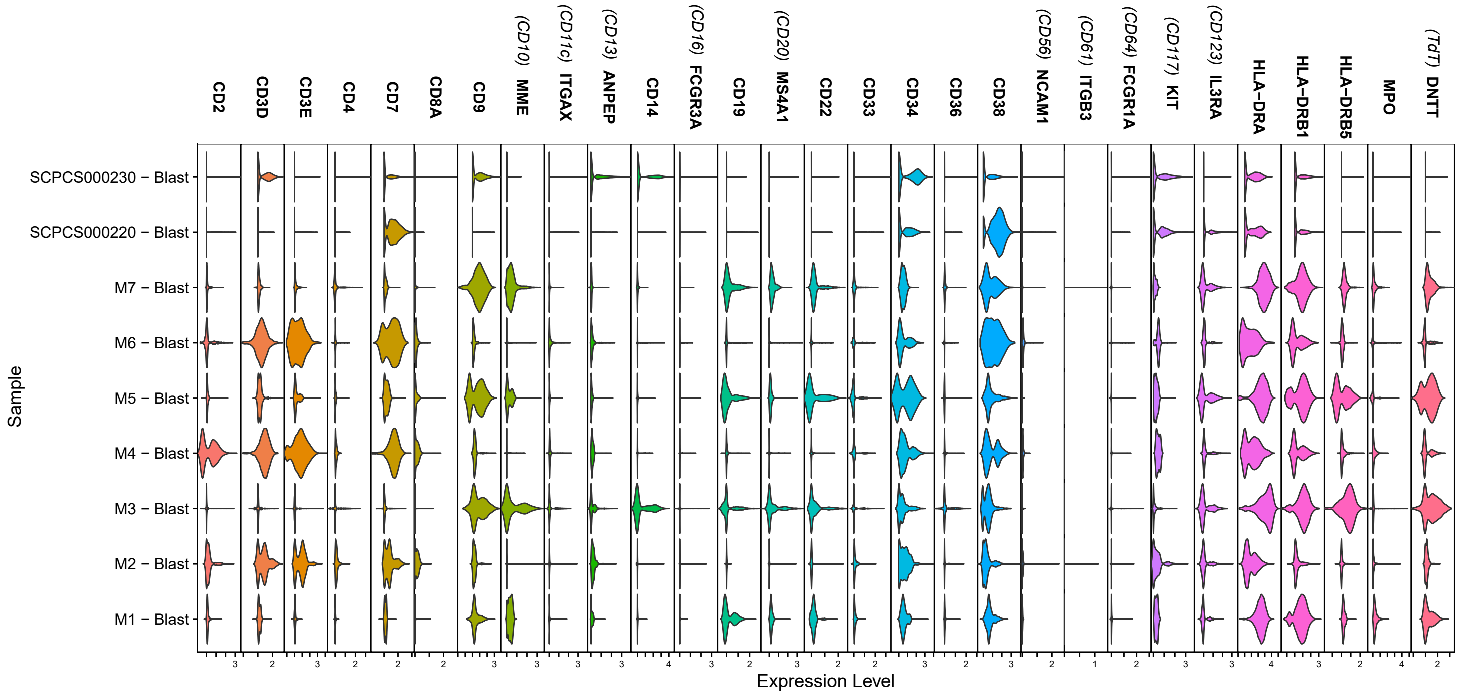

Figure S4. Single-cell RNA expression of markers used for clinical diagnosis with flow cytometry. The violin plots show the log normalized, batch corrected expression values for each flow cytometry marker (with alternative gene names) in the MPAL samples’ blast cells.

Fig S5

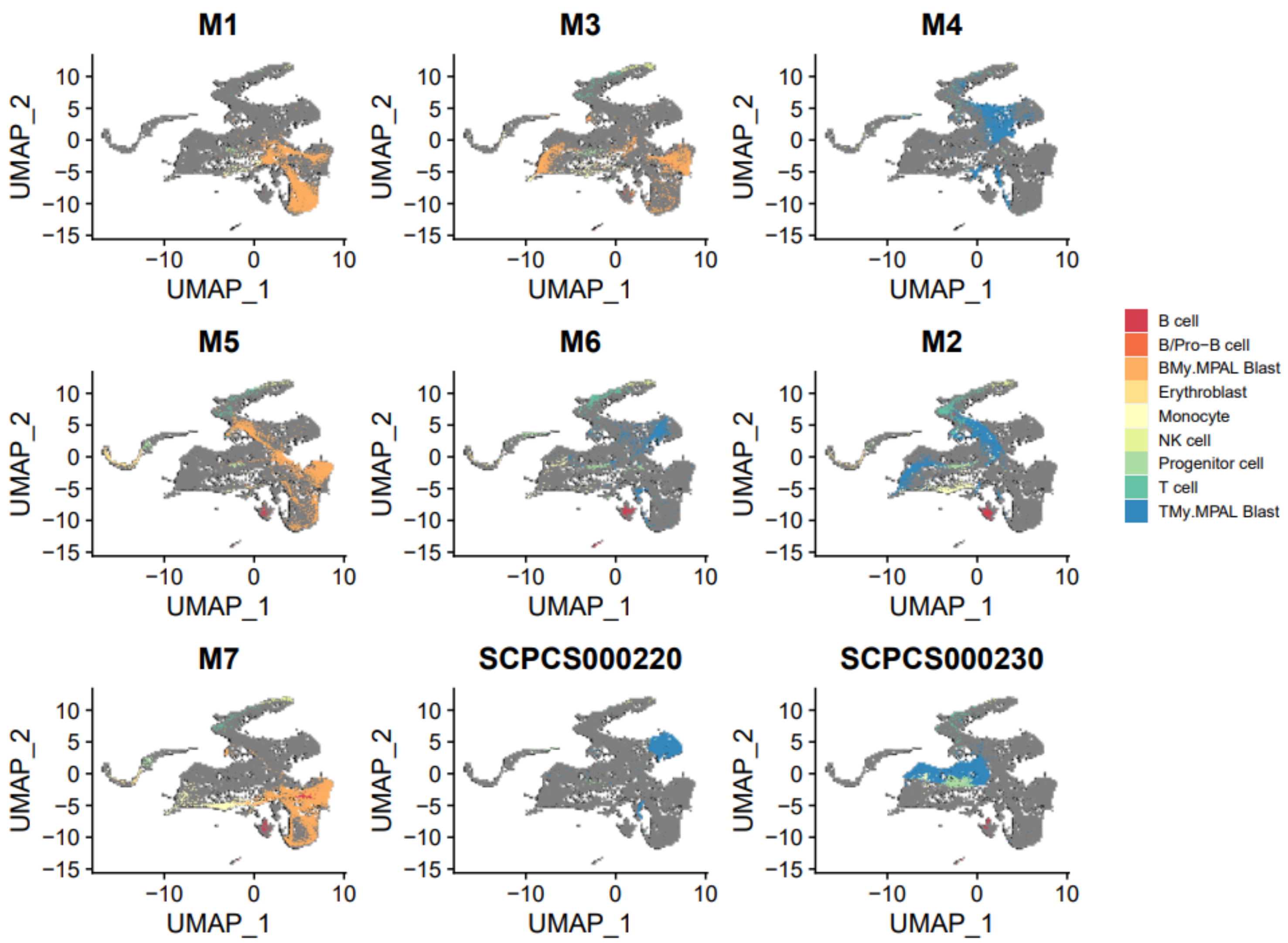

Figure S5. Feature plots highlighting cells for each MPAL sample. The cell locations of the four B/Myeloid MPAL samples (M1, M3, M5, M7) and the five T/Myeloid MPAL samples (M2, M4, M6, SCPCS000220, SCPCS000230) are highlighted in separate UMAP plots.

Fig S6

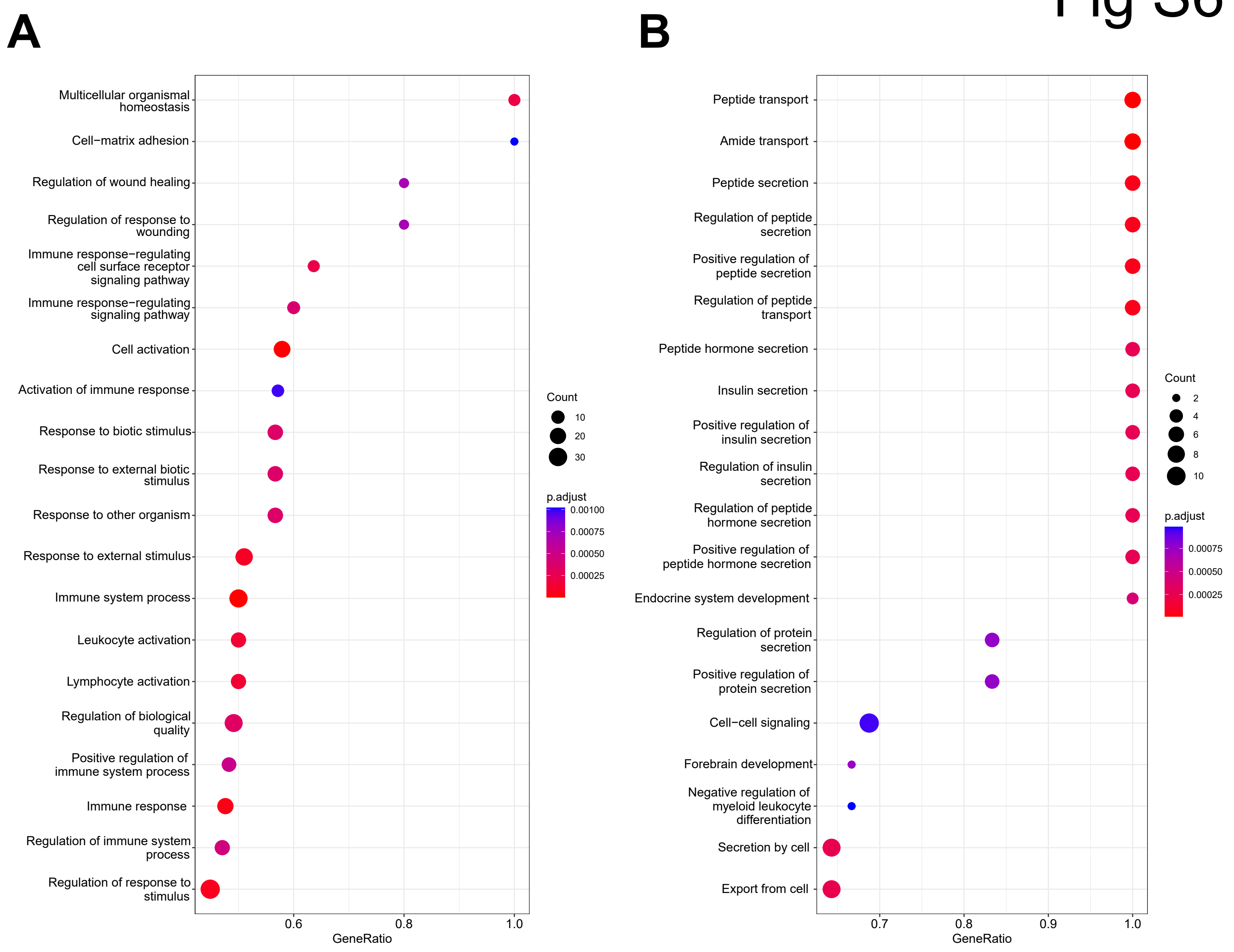

Figure S6. Enriched GO Biological Processes in the genes significantly overexpressed (avg. log2FC>0.25, p-value<0.05) in MPAL subtypes. **A)** Top 20 enriched gene sets in the B/My MPAL blast cells as compared to healthy cells. **B)** Top 20 enriched gene sets in the T/My MPAL blast cells as compared to healthy cells. The size of the dot represents the number of genes that belong to a gene-set, the Gene Ratio represents the size of the overlap between blast cells overexpressed query genes and a given gene-set, and the color of the dots represent the significance of association based on “BH” adjusted p-values.

Fig S7

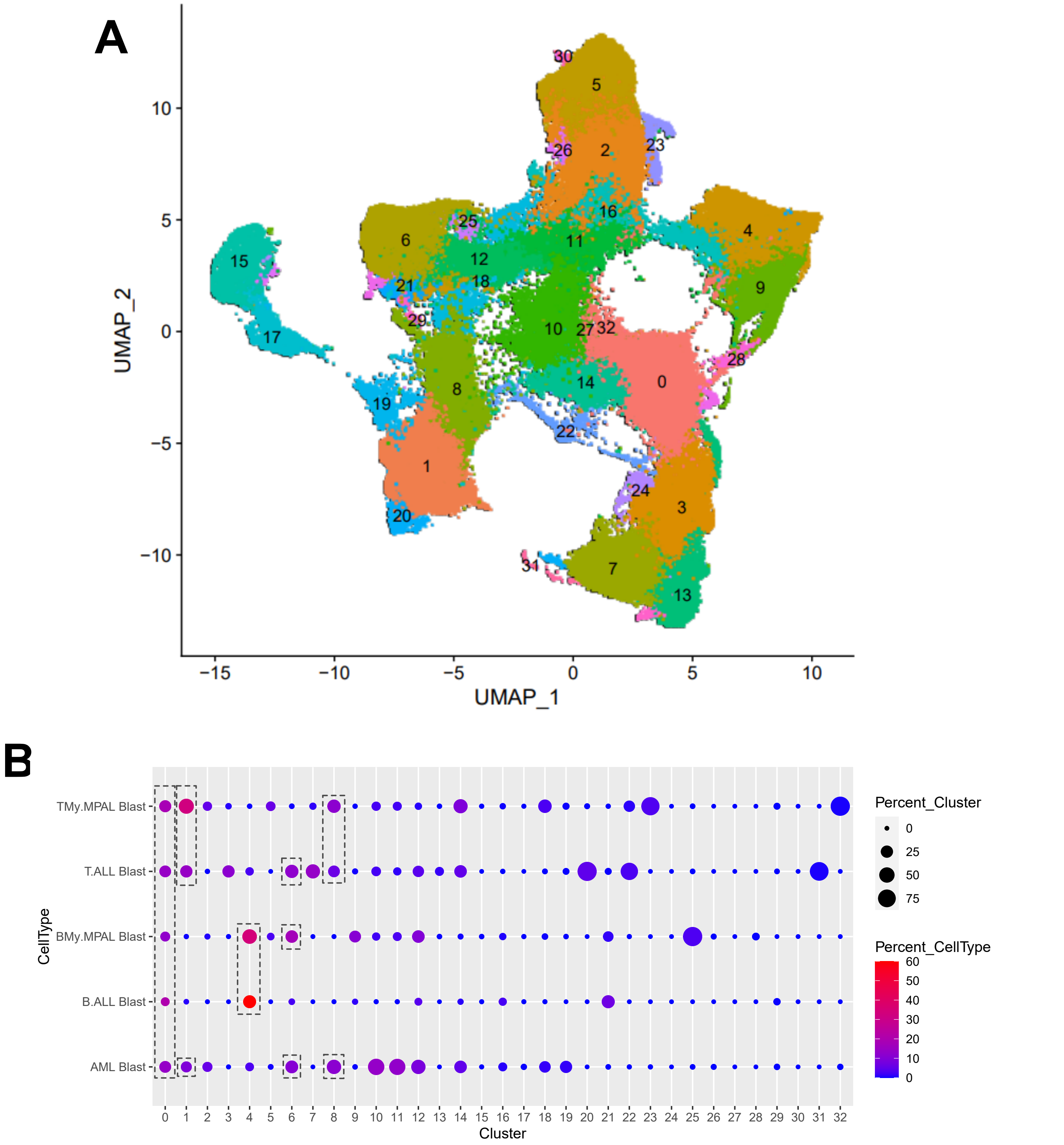

Figure S7. UMAP single-cell clusters of Acute Leukemias and healthy bone marrow cells. A) An unsupervised, KNN graph-based clustering method was applied to generate 33 distinct clusters of acute leukemias and healthy bone marrow samples. **B)** Dot plot showing the percent of each blast cell type of acute leukemia contributing toward cell clusters. The clusters of interest are shown in the grey boxes. The dot size and color represent the proportional size of the cluster (number of cells in cluster / total cells in object) and percent contribution of blast cell type toward each cluster respectively. The red and blue colors represent th e high and low contribution of cell types in the clusters respectively.

Fig S8

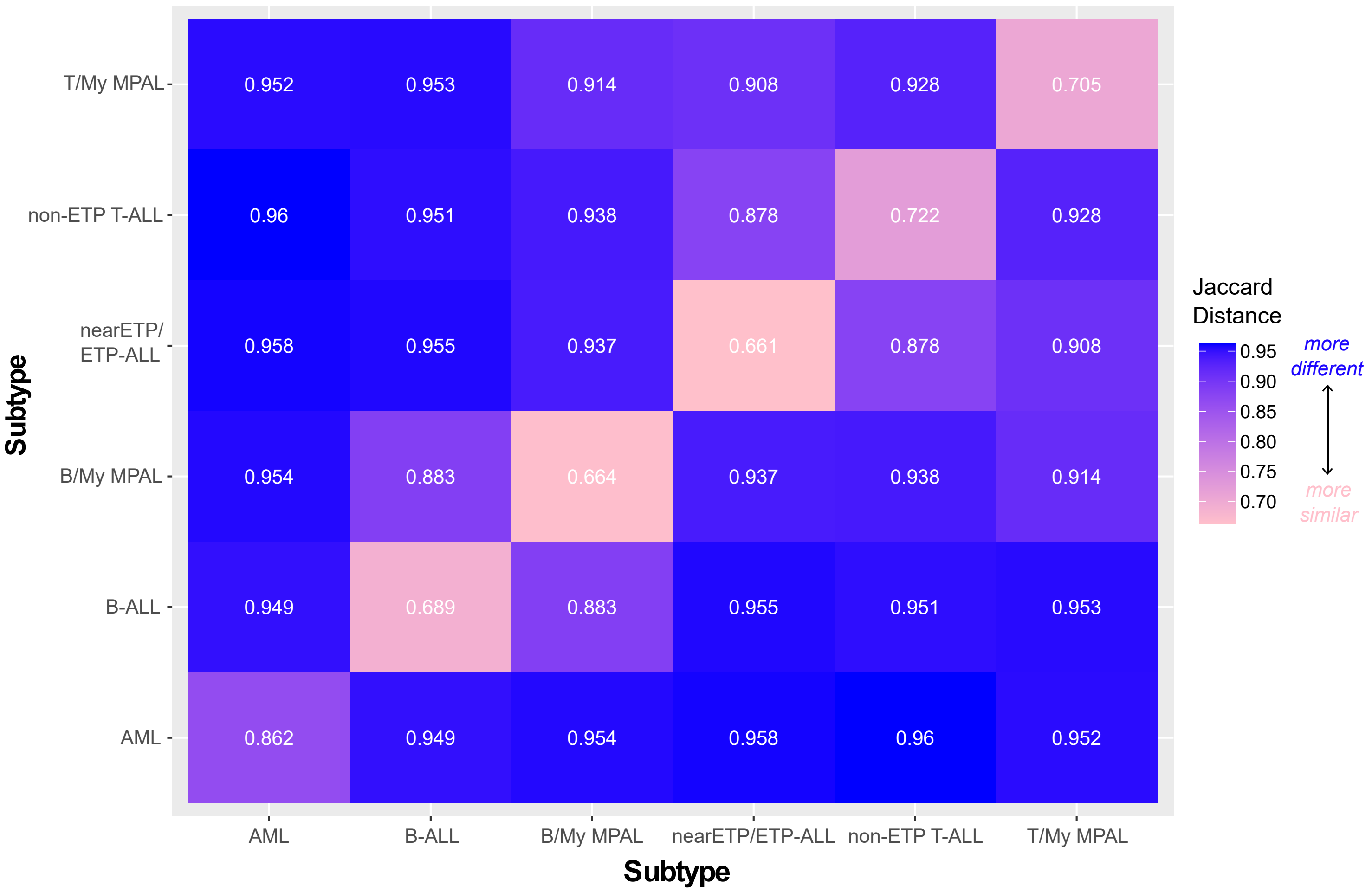

Figure S8. Average sample to sample distances between subtypes based on common over-expressed genes. The over-expressed genes (average log2FC>0.25 and adjusted p-value<0.05) were found when comparing sample blast cells to each other. To assess their differences, the Jaccard distance (1 – size of intersection divided by size of union) was calculated for each pair of samples. The average distance for the sample pairs between two subtypes was calculated and plotted on a heatmap. The blue color represents subtypes that are more different and pink represents subtypes that are more similar based on their common blast over-expressed genes between sample pairs.

Fig S9

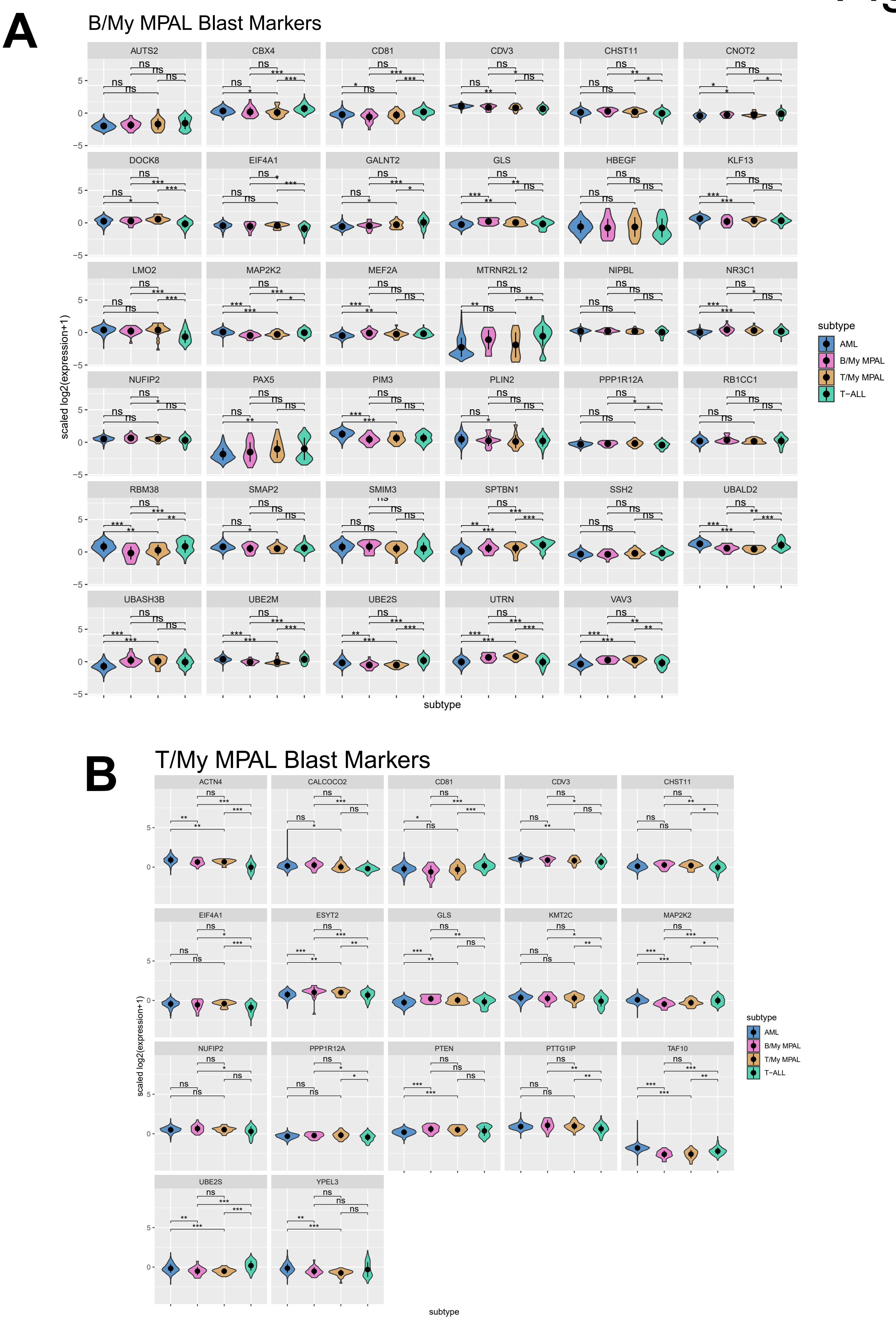

Figure S9. Expression of MPAL blast biomarker genes in the bulk RNA-Seq data. A) **Scaled** log2(expression+1) for each of the B/My MPAL blast biomarkers. **B)** Scaled log2(expression+1) for each of the T/My MPAL blast biomarkers. Transcripts per million (TPM) values for bulk RNA - Seq data were downloaded from the TARGET initiative portal (https://www.cancer.gov/ccg/research/genome-sequencing/target). The significance between groups was calculated using Wilcoxon rank tests, with "***" representing p -value<0.001, "**" representing p-value<0.01, "*" representing p-value<0.05, and "ns" representing p>0.05.

Fig S10

A

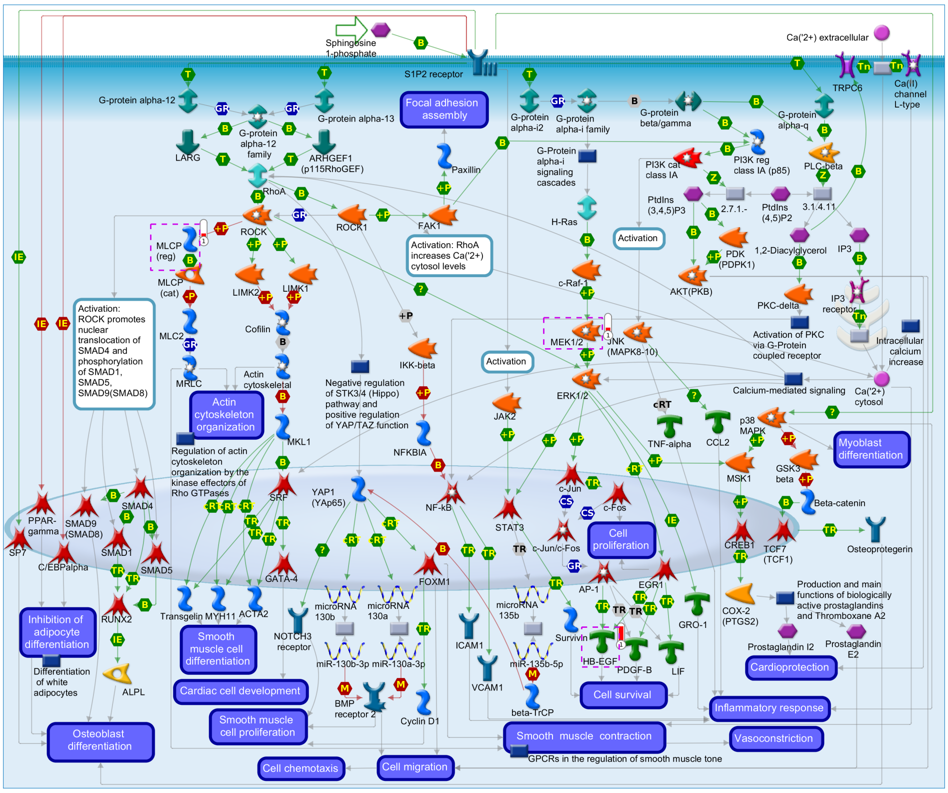

B

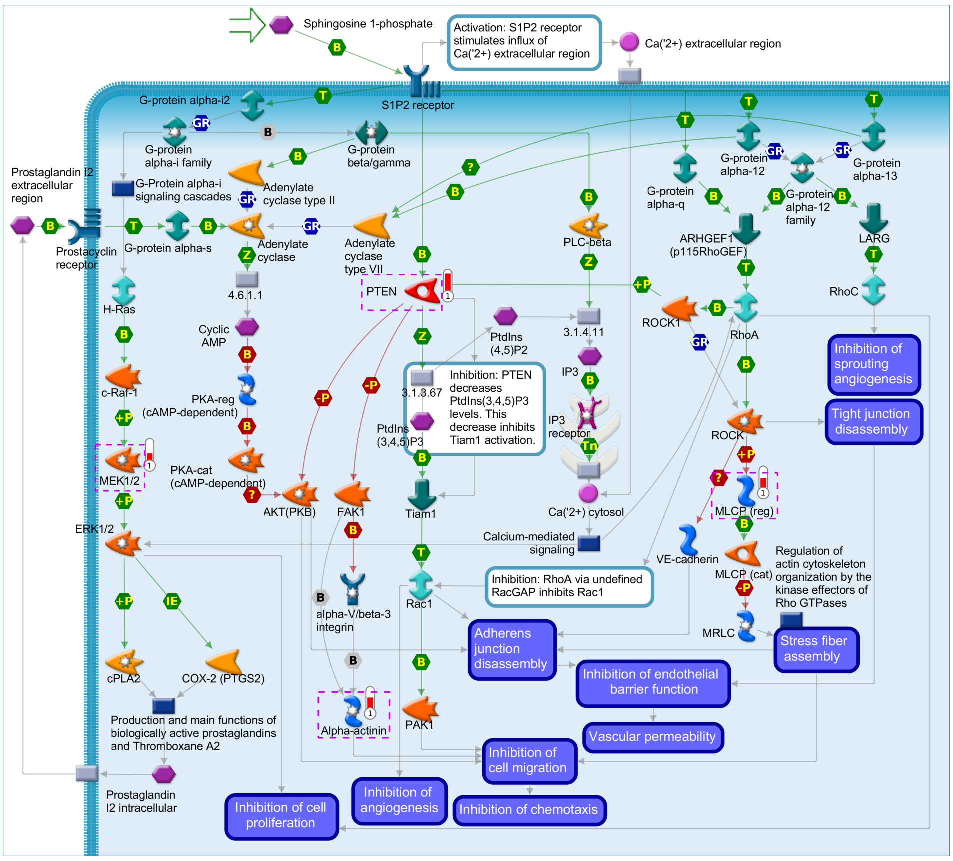

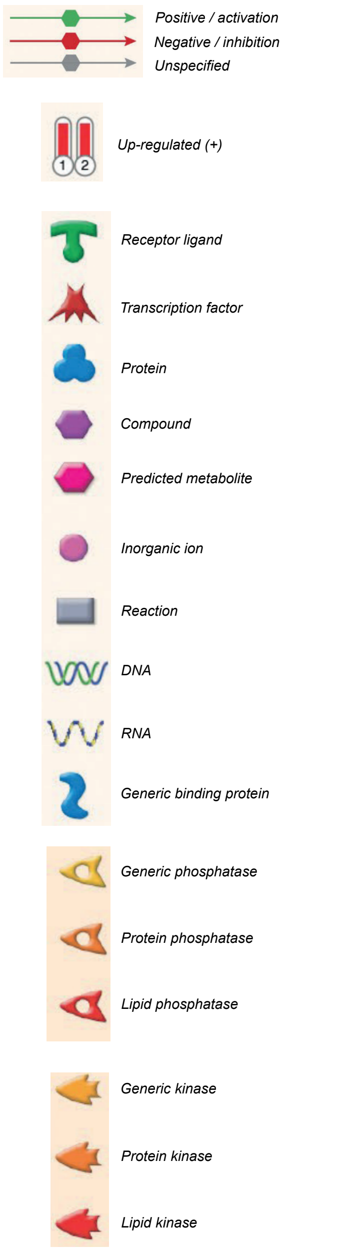

See MetaCore Website for Full Symbol Legend

Figure S10. Pathway enrichment of significantly over-expressed genes in MPAL subtypes blast cells. **A)** S1P2 receptor activation signaling pathway, with significant upregulation of HBEGF, MEK1/2 (MAP2K2), and MLCP (PPP1R12A) in the B/My MPAL biomarker set and marked with the red square. **B)** S1P2 receptor inhibitory signaling pathway, with PTEN, MLCP (PPP1R12A), MEK1/2 (MAP2K2), and Alpha-actin (ACTN4) significant upregulation in the T/My MPAL biomarker set and marked with the red square.

Fig S11

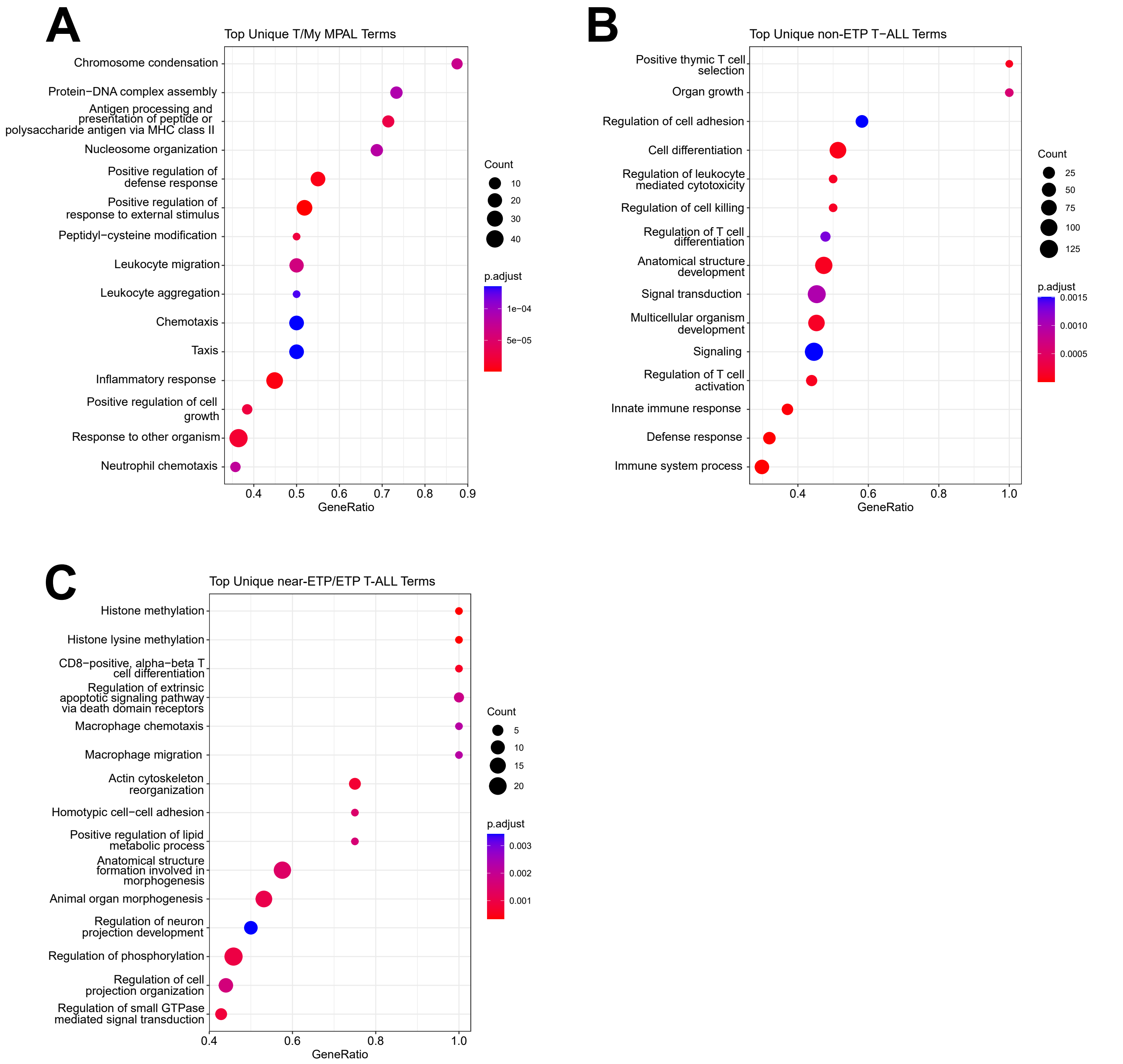

Figure S11. Top unique enriched Gene Ontology gene sets for T/My MPAL **(A)**, non-ETP T-ALL **(B)**, and near-ETP/ETP-ALL **(C)** blast marker sets. The gene ontology analysis was performed using clusterProfiler and Biological Process GO categories with Benjamini–Hochberg P value < 0.05 are considered significant.

Fig S12

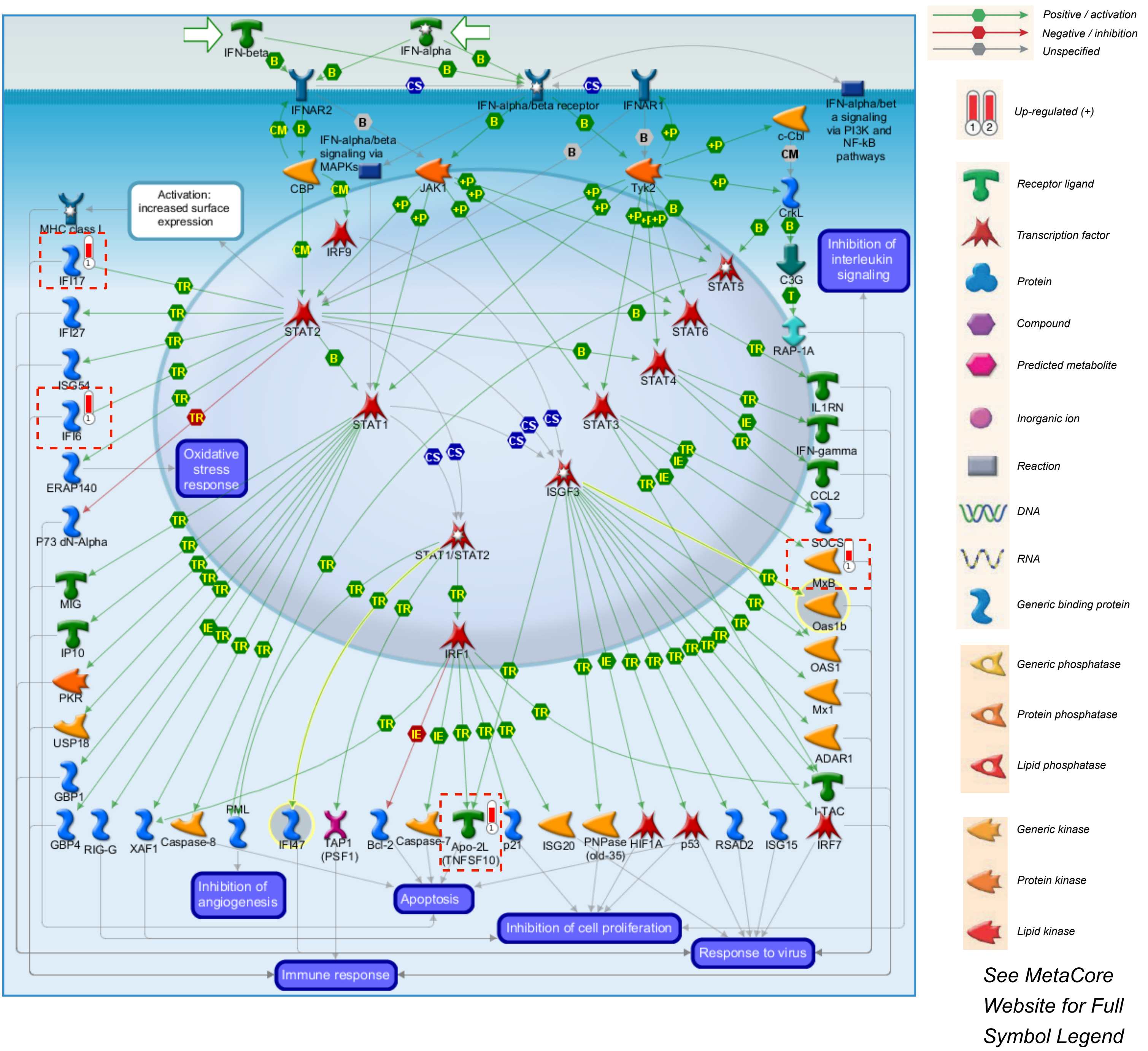

Figure S12. Detailed view of IFN-alpha/beta signaling via JAK/STAT pathway that was significantly affected (p-value<0.05) in the commonly over-expressed genes for Dx-Rel (future relapse) as compared to Dx-Rem (future remission) blast cells in both MPAL subtypes. The pathway enrichment analysis was performed using the MetaCore platform. The genes of the signaling pathway that were significantly up-regulated in the Dx-Rel blast cells are highlighted with a red dotted box.

Fig S13

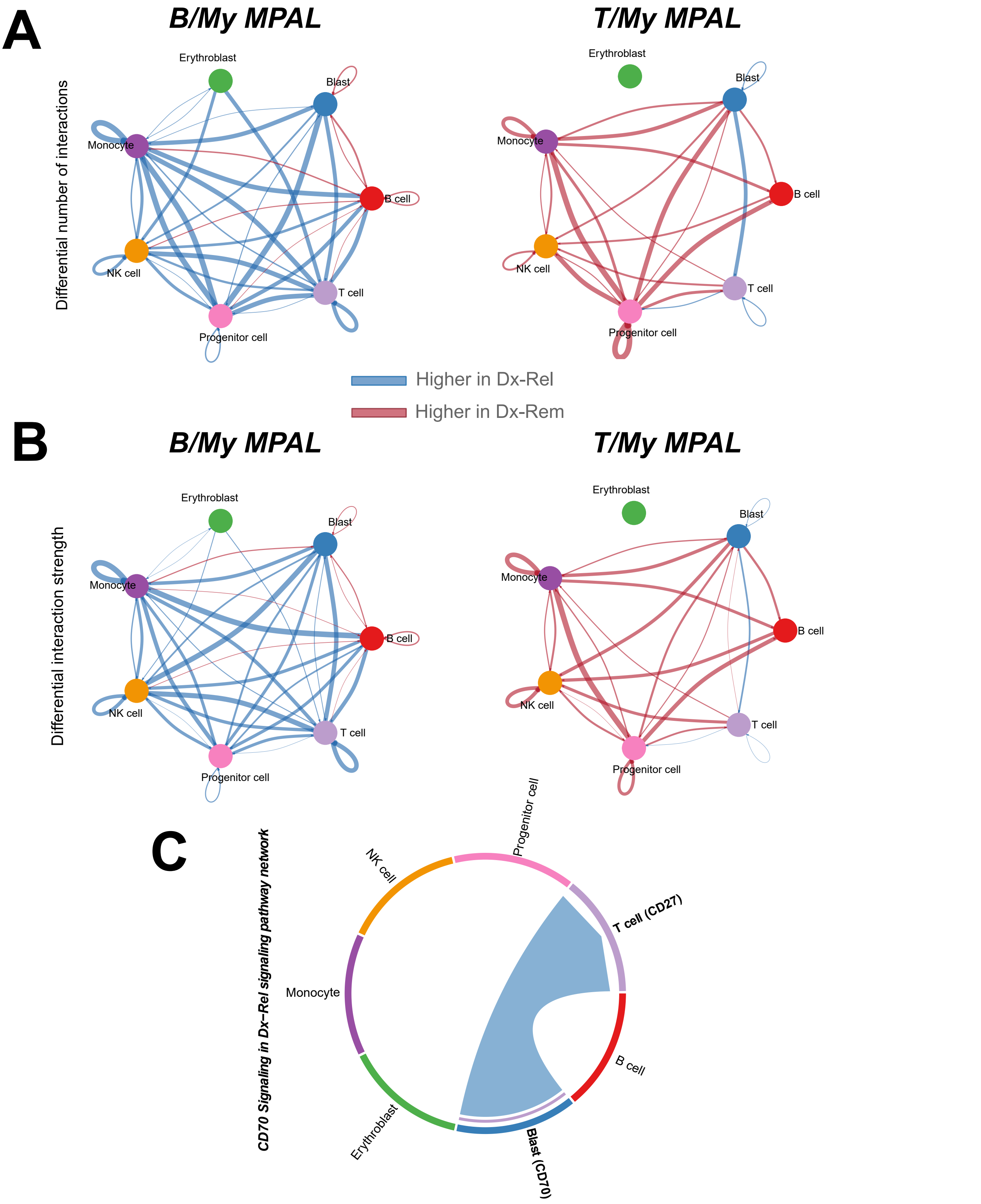

Figure S13. Cellular communication circle plots for Dx-Rel and Dx-Rem cells in MPAL subtypes. **A)** The differential number of interactions between Dx-Rel and Dx-Rem samples for each subtype. The cellular communication was estimated based on the ligand and receptor expression between interacting cell types and shown with arrows. The thickness of the arrow represents the relative number of interactions among cell types. **B)** The differential interaction strength between Dx-Rel and Dx-Rem samples. Each arrow represents the relative strength of interactions among cell types. The arrows are colored in red and blue depending on the higher number or strength interactions in Dx-Rel or Dx-Rem samples respectively. **C)** CD70 signaling in T/My MPAL Dx-Rel cells. The chord diagram shows the sender and receiver cell type, along with the ligand (CD70) and receptor (CD27).
